# Novel *Bacillus*-Infecting Phage *Bquatquinnuvirus eskimopiis* (Strains B450T and B450C), Founder of a New Genus, and the Properties of Its Endolysin

**DOI:** 10.3390/ijms27010131

**Published:** 2025-12-22

**Authors:** Olesya A. Kazantseva, Olga N. Koposova, Irina A. Shorokhova, Vladislav A. Kulyabin, Andrey M. Shadrin

**Affiliations:** G.K. Skryabin Institute of Biochemistry and Physiology of Microorganisms, Pushchino Scientific Center for Biological Research of the Russian Academy of Sciences, Federal Research Center, Prospect Nauki, 5, 142290 Pushchino, Russia; koposova@pbcras.ru (O.N.K.); shorokhovai@pbcras.ru (I.A.S.); kuliabin.vlad@pbcras.ru (V.A.K.); a.shadrin@pbcras.ru (A.M.S.)

**Keywords:** *Bacillus cereus*, *Bacillus thuringiensis*, bacteriophage, phage, prophage, endolysin

## Abstract

This study characterizes two novel *Bacillus* phages, B450T and B450C, isolated from *Bacillus thuringiensis* VKM B-450 via mitomycin C induction, along with their endolysin, PlyC19. Both phages, siphoviruses with 41,205 bp genomes, lysed 38% of the tested *Bacillus cereus* sensu lato strains, with B450C showing enhanced lytic activity due to mutations in the repressor protein. PlyC19 lysed 56% of the strains tested, including *Priestia flexa*, demonstrating broader efficacy. Its Amidase_2 domain and dual SH3 cell wall-binding domains enable targeted peptidoglycan hydrolysis, with optimal activity at pH 9.0 and thermal stability up to 40 °C. We propose the taxonomic designation *Bquatquinnuvirus eskimopiis* for these phages, with B450T and B450C representing distinct strains, based on genomic divergence in the repressor protein’s HTH_Xre domain, consistent with their turbid and clear plaque morphologies, respectively. PlyC19′s broad specificity underscores its potential as an enzybiotic against multidrug-resistant *Bacillus cereus* group strains in food safety and medicine.

## 1. Introduction

Temperate bacteriophages are capable of two distinct life cycles: lytic replication, which leads to the production of new virions; and lysogeny, in which the phage genome is usually maintained as an integrated prophage within the bacterial chromosome [1,2], although some phages (for example P1, N15, B83) exist as extrachromosomal plasmid prophages [3,4,5]. In the lysogenic state, the phage genome is stably maintained within the bacterial host and replicated as an integrated component of the cellular genome [2]. Genomic analyses indicate that approximately 50% of bacterial genomes harbor at least one prophage, while some bacterial lysogens contain up to 16 prophages [6].

In this symbiotic relationship, the phage secures a temporary niche within the host cell while retaining the potential to switch to the lytic cycle. This dynamic enables prophages to transfer genes that enhance host survival, thereby promoting mutual benefits. Consequently, temperate phages play a pivotal role in bacterial evolution by driving horizontal gene transfer, which modifies host traits such as pathogenicity, biofilm formation, stress resistance, and antimicrobial resistance [7]. For example, studies of antibiotic-treated mouse gut microbiomes demonstrate that phages disseminate resistance genes, reshaping microbial community dynamics [8]. Prophages are also well-known for encoding virulence factors that can transform benign bacteria into pathogens. Notable examples include *Escherichia coli* phages H-19A, H-19B, and 933W, which carry genes for Shiga toxins Stx1 and Stx2 [9,10]; *Corynebacterium diphtheriae* phages encoding the diphtheria toxin *tox* operon [11,12]; and the CTXφ phage of *Vibrio cholerae*, which harbors the *ctxAB* genes for cholera toxin [13].

Beyond virulence, prophages can modify bacterial surface components, such as polysaccharides or proteins, to facilitate immune evasion and complicate vaccine development. For instance, *Salmonella*’s P22 phage modifies O-antigens with glycosyl groups [14], and *Shigella*’s Sf6 phage facilitates O-acetylation [15,16]. Prophages also enhance adhesion and invasiveness; the lambda phage’s *lom* gene improves *E. coli* attachment to mucosal surfaces [17], while *Streptococcus mitis*’ SM1 phage encodes adhesins PblA and PblB for platelet binding [18].

Prophages further influence microbial community ecology by modulating biofilm formation, sporulation, and resistance to secondary infections. In *Bacillus anthracis* strain ΔSterne, prophages Bcp1, Wip4, Wip1 and Frp2 promote exopolysaccharides and biofilm development through phage-specific RNA polymerase sigma factors [19], while in *Bacillus subtilis*, phages PMB12 and SP10 overcome catabolite repression to induce sporulation [20]. Prophages encode superinfection exclusion genes, such as *Salmonella* phage P22′s *sie*A or *Pseudomonas* phage D3112’s *gp*05 (encoding Tip protein), which block DNA injection or disrupt type IV pilus assembly, respectively, thereby protecting against related phages [21,22]. Moreover, spontaneous prophage activation can provide lysogens with a competitive edge by eliminating nearby susceptible bacteria [23], though multiple prophage carriage often evolves to mitigate such effects [6].

Notably, pathogenic bacteria often harbor integrated prophages. For example, approximately 80% of *Streptococcus pneumoniae* clinical isolates carry prophages [24]. Prophage profiles are valuable for pathogen identification, strain typing, and understanding gene regulation and disease mechanisms [25]. Prophage polymorphisms have enabled differentiation of *Staphylococcus aureus* variants producing exfoliative toxin A [26], and unique prophage combinations, such as the four in *B. anthracis*, have supported targeted diagnostic tools like multiplex PCR assays [27]. However, the lysogenic nature of temperate phages often renders them unsuitable for biotechnological applications, highlighting the need to identify lysogeny-related genes. Their narrow host range limits their utility in biocontrol but enhances their diagnostic potential.

Despite the limitations of temperate phages for direct biotechnological applications as intact virions, their genomes, like those of lytic phages, encode endolysins that hold significant potential for therapeutic innovation. Endolysins are enzymes that cleave the peptidoglycan matrix of bacterial cell walls, facilitating phage progeny release during the conclusion of the lytic cycle. For phages infecting Gram-positive bacteria, such as *Bacillus*, endolysins can also disrupt cell walls when applied exogenously, enabling their use as novel antimicrobial agents to complement or replace conventional antibiotics. Compared to traditional antibiotics, endolysins exhibit higher target specificity, and studies indicate that bacterial resistance to these enzymes develops more slowly [28]. Moreover, endolysins circumvent challenges associated with whole-phage therapies, such as prophage-induced immunity, positioning them as flexible and effective tools for addressing bacterial infections in both medical and industrial contexts.

Genomic analyses indicate that *Bacillus* species may contain up to five prophages per genome [29]. Despite progress in sequencing technologies, the diversity, induction mechanisms, and ecological functions of temperate phages in *Bacillus* remain poorly understood, as the limited number of temperate phages that have been induced, isolated, and characterized hinders a comprehensive understanding of their biological roles.

In this study, we report the isolation and characterization of a novel bacteriophage species from the lysogenic strain *B. thuringiensis* VKM B-450. Through successive propagation and purification cycles, two morphologically distinct phage variants—B450T (forming turbid plaques) and B450C (forming clear plaques)—were obtained. Additionally, we purified the bacteriophage-encoded endolysin, and characterized its enzymatic activity. Both phage isolates and their endolysin exhibited lytic activity against members of the *Bacillus cereus* group. Phylogenetic and comparative genomic analyses, following the International Committee on Taxonomy of Viruses (ICTV) guidelines, support the classification of this bacteriophage species within a novel genus. We propose the taxonomic designation *Bquatquinnuvirus eskimopiis*, with B450T and B450C as representative strains.

## 2. Results

### 2.1. Phage Isolation, Plaque Morphology, Host Range and Transmission Electron Microscopy

**Figure 1:**
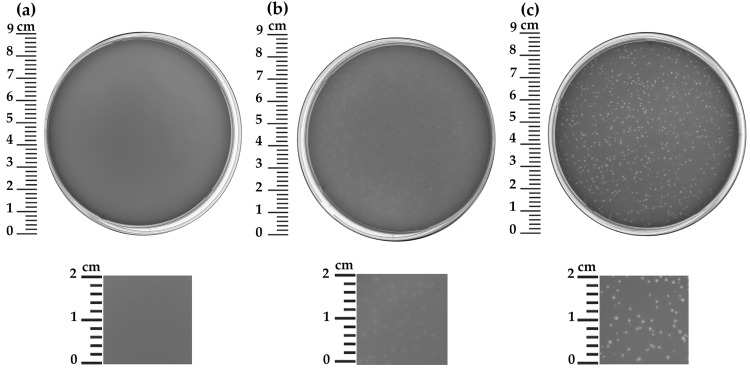
Plaque morphology of *Bacillus* phages B450T and B450C on a lawn of *B. thuringiensis* VKM B-446, prepared with 0.5% top agar. (**a**) Uninfected bacterial lawn (control); (**b**) B450T turbid plaques; (**c**) B450C clear plaques.

The phage was isolated from a lysogenic bacterial strain, *B. thuringiensis* VKM B-450, through induction with mitomycin C. During the purification of the induced phage through successive cycles of extraction and propagation, two distinct phage preparations were obtained that produced different types of plaques on a lawn of *B. thuringiensis* strain VKM B-446 (Figure 1). Both clear and turbid plaques exhibited a smooth edge and a diameter of approximately 0.5–1 mm. The isolated phage preparations were designated as B450, reflecting their origin from the host strain *B. thuringiensis* VKM B-450. To distinguish between the preparations based on their plaque morphology, the suffixes “C” and “T” were assigned to indicate clear and turbid plaques, respectively.

The host range of *Bacillus* phages B450T and B450C was determined by testing their lytic activity against 45 bacterial strains, including members of the *Bacillus* genus and species of the genus *Priestia* (previously classified under *Bacillus*), such as *Priestia flexa* and *Priestia megaterium*. The phages successfully lysed 17 strains (approximately 38%), exclusively infecting species *B. cereus* sensu stricto and *B. thuringiensis*. This specificity highlights the narrow host range of B450T and B450C, targeting selected members of the *B. cereus* group. Detailed results of the host range analysis are provided in Supplementary Information, Table S1.

**Figure 2:**
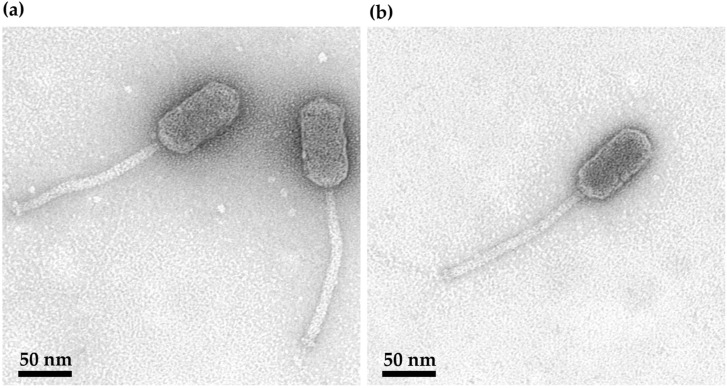
TEM characterization of phage virions. B450T (**a**) and B450C (**b**) particles negatively stained with uranyl acetate. (1% *w*/*v* uranyl acetate). Scale bar: 100 nm. The full-TEM micrographs are available in Supplementary information, Figures S1 (B450T) and S2 (B450C).

Transmission electron microscopy (TEM) analysis (Figure 2) revealed that the B450T and B450C virions share the same morphological features, characteristic of the siphovirus morphotype. The virions possess an elongated capsid measuring 86.4 ± 1.5 nm in length and 40.7 ± 1.1 nm in diameter. Additionally, they feature a non-contractile tail with a length of 152.2 ± 1.8 nm and a diameter of 10.6 ± 0.5 nm.

### 2.2. Killing Assay of Bacteriophages

**Figure 3:**
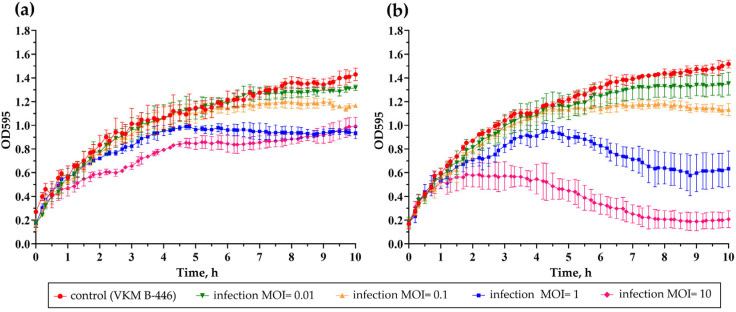
Growth kinetics of *B. thuringiensis* VKM B-446 infected with phages B450T (**a**) and B450C (**b**) at varying MOI values. Bacterial growth curves were monitored after phage infection at MOIs of 0.1, 1, and 10, as indicated in the legend. Data points represent mean optical density (OD595) values from three independent trials ± standard deviation. Graphs were generated using GraphPad Prism 8.4.3 for data visualization.

The growth dynamics of *B. thuringiensis* strain VKM B-446 infected with phages B450T and B450C at varying multiplicities of infection (MOI) was assessed by measuring the optical density at 595 nm (OD595) of infected cultures and comparing these to an uninfected control culture. The resulting bacterial growth curves are presented in Figure 3. 

B450T (Figure 3a) exhibited lytic activity characteristic of temperate phages, with incomplete lysis of the sensitive bacterial culture even at the highest MOI tested (MOI = 10). In contrast, B450C displayed a lytic pattern typical of virulent phages, where lytic activity increased with higher MOI values, culminating in complete inhibition of bacterial growth at an MOI of 10 (Figure 3b).

### 2.3. Thermal and pH Stability Assay of Bacteriophages

**Figure 4:**
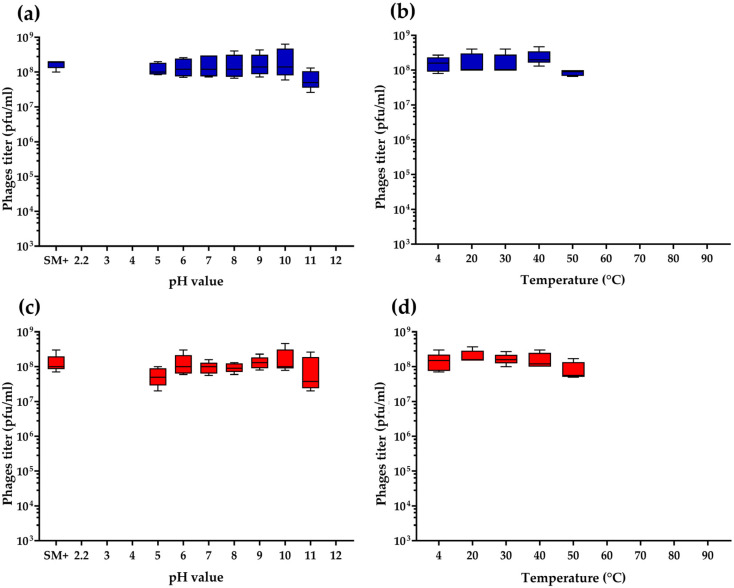
Stability profiles of Bacillus phages B450T (**a**,**b**) and B450C (**c**,**d**): pH stability (**a**,**c**) and thermal stability (**b**,**d**). The box-and-whisker plots are shown in blue for phage B450T (panels **a**,**b**) and in red for phage B450C (panels **c**,**d**). Data represent mean viability from five biological trials ± standard deviation. Graphs were generated using GraphPad Prism 8.4.3 for data visualization.

To investigate the stability of *Bacillus* phages B450T and B450C, their resilience to varying pH and temperature conditions was evaluated. Both phages demonstrated high stability across a pH range of 5 to 11, maintaining consistent titers comparable to control samples (SM+ buffer) incubated at pH = 7.5 (Figure 4a,c). Similarly, both B450T and B450C exhibited robust thermal stability at temperatures ranging from 20 °C to 50 °C, with phage titers remaining equivalent to those of control samples incubated at 4 °C (Figure 4b,d). After 1 h incubation at 60 °C and higher (60, 70, 80 and 90 °C), no infectious particles were detected for either phage.

### 2.4. Genome Characteristics

#### 2.4.1. General Genome Organization

**Figure 5:**
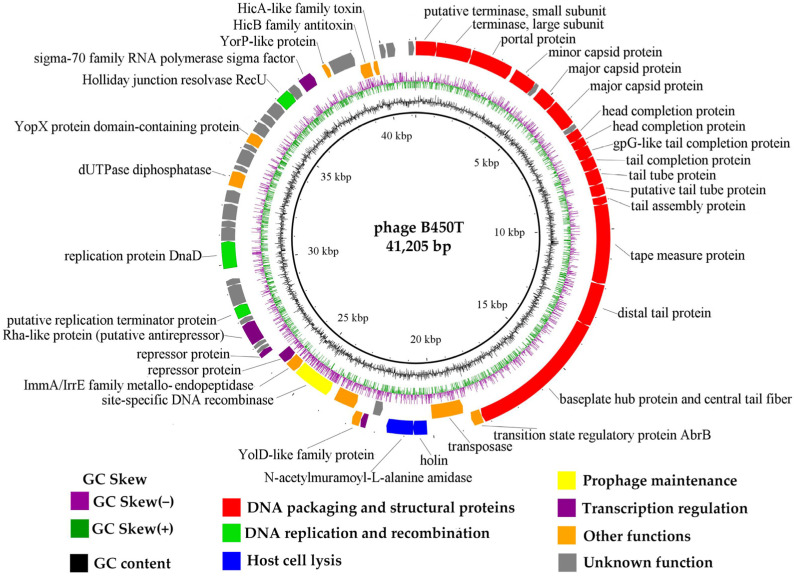
Circular genome map of *Bacillus* phage B450T. Predicted ORFs are color-coded according to their functional annotation categories, as indicated in the legend. Key genomic features are indicated.

DNA of both *Bacillus* phages B450T and B450C was extracted and sequenced to elucidate their genetic structure and functional organization. The genomes consist of linear double-stranded DNA, each spanning 41,205 bp with a GC content of 34.9%. Comparative analysis revealed that the genomes differ solely in the sequence of gene 29 (Supplementary Information, Table S2), which encodes a repressor protein, with two amino acid substitutions potentially modulating the lysis–lysogeny switch. The near-identical genomes of B450T and B450C indicate they likely originate from the same prophage in VKM B-450, with differences arising from mutations in the repressor gene during induction or propagation. Future sequencing of the host genome could confirm integration sites and reveal additional prophages. Given the near-identical sequences of the two phage strains, B450T was selected as the representative for detailed analysis. The B450T genome comprises 60 predicted open reading frames (ORFs), of which 28 (46.7%) were functionally annotated using BLASTp and HHpred. The functional categories included DNA packaging, structural proteins, replication and recombination, lysis, regulatory functions, and others. A circular genome map of B450T, with the first base of the small terminase subunit gene as the starting point, illustrates the arrangement of the predicted ORFs, and other genomic features (Figure 5). Detailed ORF annotations are provided in Supplementary Information, Table S2.

#### 2.4.2. DNA Packaging Genes and the Genome Packaging Strategy

**Figure 6:**
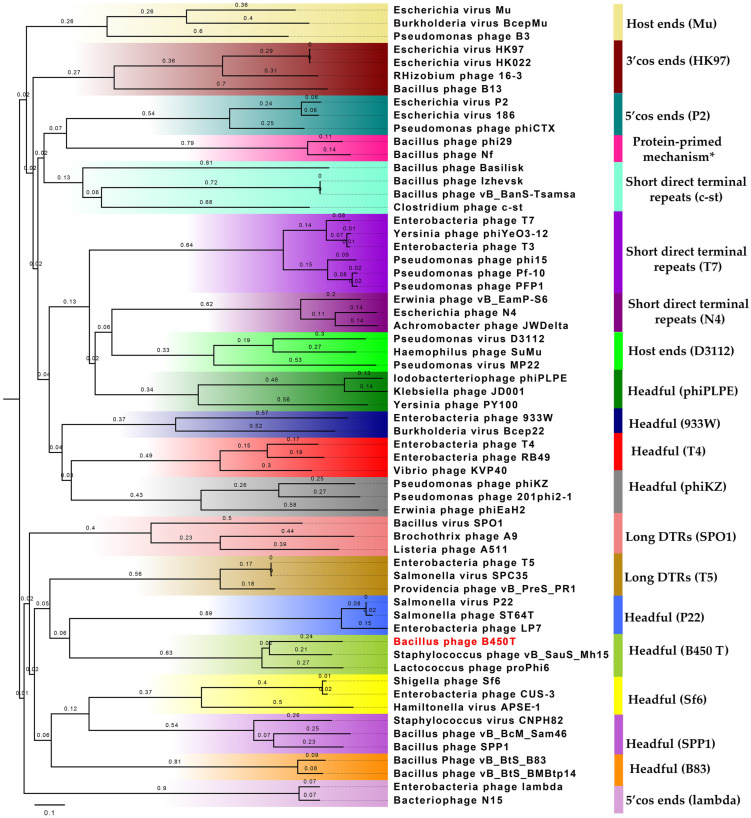
Phylogenetic analysis of large terminase subunits from Bacillus phage B450T (indicated in red) and related phages with characterized packaging strategies. The evolutionary tree was reconstructed using the Neighbor-Joining method (MEGA X) with 1000 bootstrap replicates. Branch support values are shown, and the tree was visualized in FigTree v1.4.4.

The DNA packaging module of *Bacillus* phages B450T and B450C comprises small terminase subunits (B450T: protein ID XYL31757.1; B450C: protein ID XYL31697.1) and large terminase subunits (B450T: protein ID XYL31758.1; B450C: protein ID XYL31698.1). Bioinformatic analysis revealed that B450T and B450C employ the headful mechanism for DNA packaging. The large terminase subunits from B450T and B450C cluster within a clade alongside closely related large terminase subunits from *Staphylococcus* phage vB_SauS_Mh15 (UKM36587.1) and *Lactococcus* phage proPhi6 (QGJ84672.1), both known to utilize the headful DNA packaging mechanism (Figure 6). The evolutionary tree, reconstructed based on the large terminase subunits using the Neighbor-Joining method in MEGA X with 1000 bootstrap replicates and visualized in FigTree v1.4.4, is presented in Figure 6 (see Supplementary Information, Table S3 for the list of large terminase subunit proteins used for phylogenetic inference).

**Figure 7:**
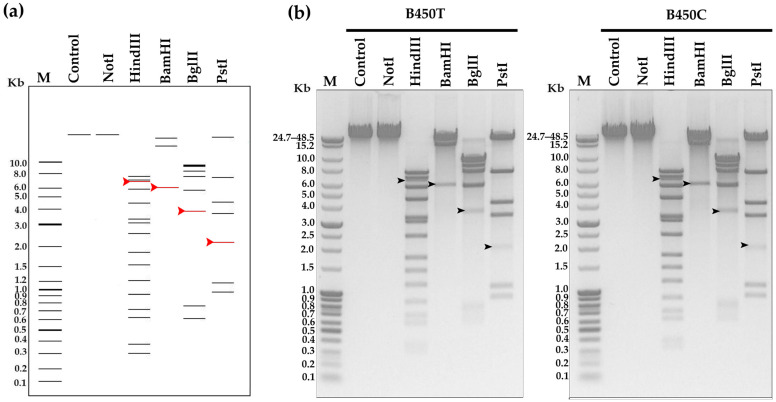
Determination of the phage DNA packaging strategy. (**a**) In silico and (**b**) in vitro restriction analysis of bacteriophage DNA. In silico digestion was performed using NEBcutter V2.0. M—molecular weight markers; control—uncut phage DNA. Fragments containing pac-sites are indicated by red and black arrows. The complete gel image is available in Supplementary Information, Figure S3.

Restriction analysis of B450T and B450C genomic DNA, conducted both in silico using NEBcutter V2.0 and in vitro with restriction endonucleases BamHI, HindIII, BglII, and PstI, confirmed the headful packaging mechanism with site-specific initiation. Electrophoretic analysis revealed distinct pac-fragments, indicative of terminase-mediated cleavage during the initial packaging event at genomic concatemers. These fragments, marked by red arrows in the in silico analysis (Figure 7a) and black arrows in the in vitro analysis (Figure 7b), were consistently observed across digests, supporting the presence of a pac-site. The complete gel image is provided in Supplementary Information, Figure S3. These findings collectively confirm that B450T and B450C utilize a headful DNA packaging strategy with precise site-specific initiation.

#### 2.4.3. Structural, Morphogenesis and Lytic Genes

The structural and morphogenesis module of *Bacillus* phages B450T and B450C encodes proteins critical for virion assembly and host interaction. Due to the near-identical genomic sequences of B450T and B450C, ORF and protein ID numbers are provided only for B450T as the representative phage. Capsid assembly involves the portal protein (ORF 3, protein_id XYL31759.1), which forms the DNA packaging channel; the minor capsid protein (ORF 4, XYL31760.1), which enhances capsid stability; and two distinct major capsid proteins (ORF 6, XYL31762.1; ORF 7, XYL31763.1), constituting the primary framework of the elongated capsid. Two head completion proteins (ORF 9, XYL31765.1; ORF 10, XYL31766.1) stabilize the capsid and facilitate tail attachment during morphogenesis.

Tail formation is mediated by a suite of proteins, including a gpG-like tail completion protein (ORF 11, XYL31767.1) and another tail completion protein (ORF 12, XYL31768.1), which connect the tail to the capsid; a tail tube protein (ORF 13, XYL31769.1) and putative tail tube protein (ORF 14, XYL31770.1), constructing the tail’s tubular framework; the tail assembly protein (ORF 15, XYL31771.1), guiding tail assembly; a tape measure protein (ORF 16, XYL31772.1), defining tail length; a distal tail protein (ORF 17, XYL31773.1), forming part of the tail tip; and a baseplate hub protein and central tail fiber (ORF 18, XYL31774.1), responsible for host attachment. This gene arrangement is typical of siphoviruses. The central tail fiber likely possesses enzymatic activity to degrade the host peptidoglycan layer, facilitating DNA injection.

The lytic system includes proteins critical for host cell disruption to release progeny virions. The holin (ORF 21, XYL31777.1) creates membrane pores, allowing the N-acetylmuramoyl-L-alanine amidase (ORF 22, XYL31778.1) to access and degrade the peptidoglycan by cleaving the bond between N-acetylmuramic acid and L-alanine, leading to cell lysis. These lytic proteins show promise for antimicrobial applications against *Bacillus* species. In this study, the activity, stability, and specificity of the endolysin from the investigated phages were characterized, and the results are presented in the following sections.

#### 2.4.4. Replication and Recombination Genes

The annotated genes within replication and recombination module include the putative replication terminator protein (ORF 35, XYL31791.1), which likely regulates the termination of DNA replication by binding specific terminator sites; the replication protein DnaD (ORF 38, XYL31794.1), involved in initiating DNA replication by facilitating the assembly of the replication complex; and the Holliday junction resolvase RecU (ORF 51, XYL31807.1), which resolves Holliday junctions during recombination, ensuring proper segregation of replicated DNA.

#### 2.4.5. Prophage Maintenance Genes and Lysis–Lysogeny Switch

In the genomes of *Bacillus* phages B450T and B450C, genes critical for sustaining the lysogenic state and regulating the lysis–lysogeny switch were identified. The prophage maintenance module encompasses a site-specific DNA recombinase (ORF 27, XYL31783.1), encoded on the negative DNA strand, which likely facilitates integration of the phage genome into the host chromosome during lysogeny.

The lysis–lysogeny switch is mediated by two transcriptional regulators: a repressor protein (ORF 29, XYL31785.1), located upstream of the recombinase in the same orientation and likely functioning analogously to the CI repressor to promote lysogeny, and another transcriptional regulator (ORF 30, XYL31786.1), encoded on the positive strand, which likely acts as a Cro-like protein to favor lytic development. The presence and organization of these genes indicate that B450T and B450C are temperate phages capable of switching between lytic and lysogenic cycles, enabling adaptation to varying host conditions.

Genetic differences between B450T and B450C were identified in the transcriptional repressor gene (ORF 29, XYL31785.1). Specifically, B450T and B450C exhibit mutations in this gene, resulting in codon substitutions GAC to GTC and TTA to ATA, respectively. These correspond to amino acid substitutions Asp9Val and Leu10Ile, located within the HTH_Xre domain of the repressor protein. These mutations likely contribute to the observed differences in plaque morphology and lytic activity. B450T forms turbid plaques on lawns of sensitive *Bacillus* strains and exhibits lytic activity typical of temperate phages, with incomplete lysis of the bacterial culture even at a MOI of 10 (Figure 1 and Figure 3). In contrast, B450C produces clear plaques and displays lytic activity characteristic of virulent phages, achieving complete inhibition of bacterial growth at an MOI of 10 (Figure 1 and Figure 3). The amino acid substitutions in the HTH_Xre domain of B450C’s repressor protein likely impair its ability to effectively repress lytic genes, potentially disrupting the lysogenic pathway and causing B450C to predominantly follow a lytic cycle.

#### 2.4.6. Type II Toxin-Antitoxin System HicAB

A distinctive genetic feature of *Bacillus* phages B450T and B450C is the presence of a type II HicAB toxin-antitoxin system encoded within their genomes. This system, located on the negative DNA strand, comprises two genes encoding the HicA-like family toxin (ORF 57, XYL31813.1) and the type II toxin-antitoxin system HicB family antitoxin (ORF 56, XYL31812.1), with the toxin gene positioned upstream of the antitoxin gene. The HicA-like toxin gene spans 192 base pairs, encoding a 64-amino-acid protein, while the HicB-like antitoxin gene spans 426 base pairs, encoding a 142-amino-acid protein. The HicAB system likely contributes to phage maintenance or host manipulation by regulating cellular processes, potentially stabilizing the lysogenic state or influencing host survival under stress conditions.

### 2.5. Comparative Genomics

**Figure 8:**
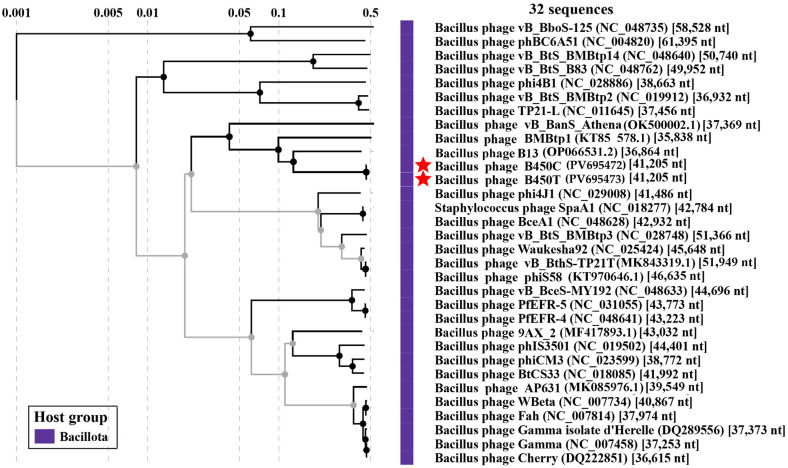
Evolutionary relationships of phages B450T and B450C. The proteomic tree was constructed using ViPTree 3.1 based on whole-genome protein similarity. Red stars indicate phages B450T and B450C.

To elucidate the phylogenetic relationships of *Bacillus* phages B450T and B450C with the known viruses, a viral proteomic tree was constructed using the ViPTree server version 3.1 (Figure 8). The analysis revealed that B450T and B450C are significantly divergent from their closest relatives. The most closely related phage was *Bacillus* phage B13, previously isolated and characterized by our group [30], sharing 37% whole-genome nucleotide identity and 42.5% of common proteins with B450T and B450C. Detailed information on whole-genome identity and the number of shared proteins for the 30 closest related phages is provided in Supplementary Information, Table S4.

**Figure 9:**
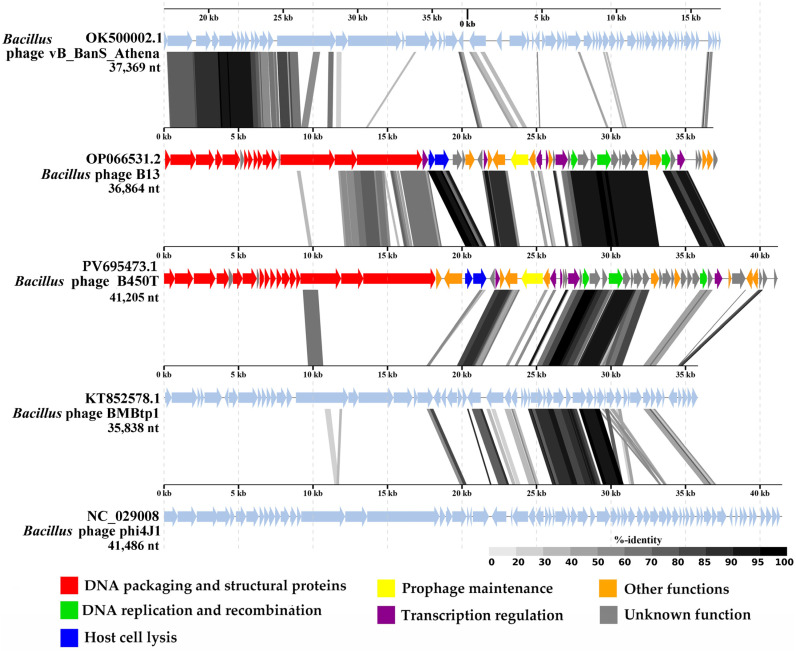
Comparative genomic analysis of phage B450T and the closest relatives. Whole-genome tBLASTx comparisons were performed using ViPTree v3.1. Proteins are color-coded by functional categories (see legend). Intergenomic similarity is represented by gray gradient plots, where shading intensity corresponds to sequence identity levels ranging from 0 to 100% (scale at right).

Pairwise tBLASTx comparison (Figure 9) identified regions of notable similarity (identity ≥ 40%) between the genomes of B450T, B450C, and B13, particularly in genes encoding replication and recombination proteins, certain transcriptional regulators, and the N-acetylmuramoyl-L-alanine amidase (endolysin), which exhibited high identity (>80%). Additionally, moderate identity (<80%) was observed in genes encoding structural proteins, including the baseplate hub protein, central tail fiber, and distal tail protein.

### 2.6. Phylogenetic Analysis and Domain Structure Analysis of Endolysin

An InterProScan analysis of the endolysin PlyC19 from *Bacillus* phages B450T and B450C revealed a domain architecture consisting of one Amidase_2 enzymatically active domain (EAD) and two cell-wall binding domains (CBDs) of the SH3 type.

**Figure 10:**
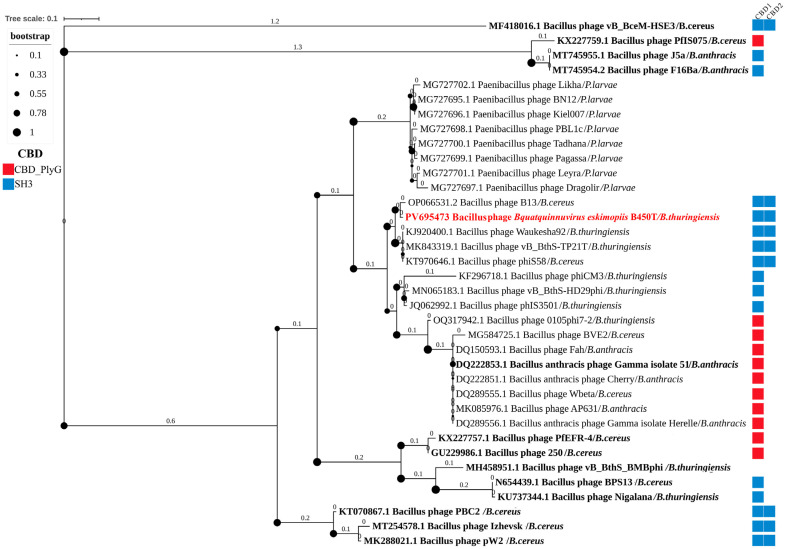
Phylogenetic tree of Amidase_2 domains of endolysins from bacteriophages infecting *Bacillus* species. The endolysin PlyC19 is highlighted in red. EADs of previously described endolysins are shown in bold. Host bacteria of the bacteriophages harboring these endolysins are indicated in italics following a forward slash (/) Red and blue squares indicate CBD_PlyG and SH3 types of CBDs, respectively.

To position PlyC19 relative to other *Bacillus* phage endolysins, a phylogenetic tree was constructed based on the amino acid sequences of Amidase_2 EADs, building on a previously established phylogenetic framework [31]. The analysis focused on a specific branch of the Amidase_2 EAD tree that includes PlyC19, encompassing EADs from phages infecting *Paenibacillus larvae*, *B. cereus*, *B. anthracis*, and *B. thuringiensis*, with CBDs of SH3 or CBD_PlyG types. The tree also incorporated Amidase_2 EADs from experimentally characterized *Bacillus* phage endolysins (Figure 10).

Phylogenetic analysis revealed that the EAD of PlyC19 exhibits high similarity to the EADs of endolysins from *Bacillus* phages B13, Waukesha92, vB-BthS_TP21T, and phiS58. Among experimentally characterized endolysins, the EAD of PlyG from *Bacillus* phage Gamma is the closest to PlyC19 in the phylogenetic tree, while other characterized EADs are more distantly related (Figure 10). BLASTp analysis of the full amino acid sequence of PlyC19 showed 94% identity with the endolysins of phages Waukesha92, vB-BthS_TP21T, and phiS58, and 87% identity with the endolysin of phage B13. These endolysins remain experimentally uncharacterized. In the constructed phylogenetic tree (Figure 10), their EADs form a distinct branch with two nodes: one comprising the EADs of endolysins from phages B450 and B13, and the other including the EADs of Waukesha92, vB-BthS_TP21T, and phiS58. Among experimentally characterized *Bacillus* phage endolysins, the highest full-sequence identity to PlyC19 is observed with the endolysin of phage pW2 (53%), although its EAD is not the closest in the phylogenetic tree [32].

**Figure 11:**
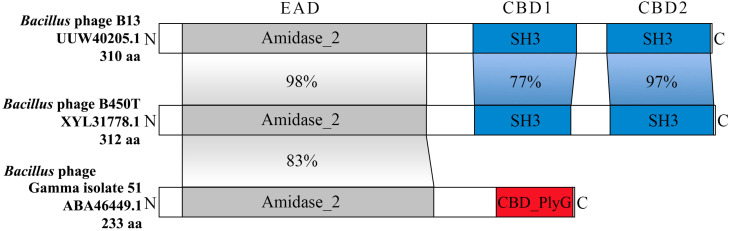
Schematic comparison of endolysin structures. Percent identity for pairwise comparisons of corresponding domains is indicated, calculated using BLASTp.

BLASTp analysis of the individual domains showed that the EAD of PlyC19 shares 98% identity with the EAD of the endolysin from *Bacillus* phage B13 and 83% identity with the experimentally characterized EAD of PlyG from *Bacillus* phage Gamma [33] (Figure 11). Structurally, the endolysin of phage B13 mirrors PlyC19, comprising one Amidase_2 EAD and two SH3 CBDs, whereas PlyG contains a single CBD_PlyG instead of two SH3 domains.

The two CBDs of PlyC19 (PlyC19 CBD1 and PlyC19 CBD2) exhibit 51% identity to each other. The PlyC19 CBD1 shares 77% identity with the CBD1 of the B13 endolysin and 98% identity with the CBD1 of the vB_BthS_TP21T endolysin. Among experimentally characterized CBDs, PlyC19 CBD1 shares 76% identity with the CBD2 of the endolysin from *Bacillus* phage Deep-Blue, notably a peptidase-class endolysin rather than an amidase [34]. The PlyC19 CBD2 is 97% identical to CBD2 of the B13 endolysin; however, among experimentally characterized CBDs, it shares only 55% identity with the CBD1 of the endolysin from *Bacillus* phage PBC2 [35].

### 2.7. Lytic Spectrum of Endolysin

The endolysin PlyC19 was successfully obtained in a purified in electrophoretically homogeneous form, with electropherogram illustrating protein overproduction and purification provided in Supplementary Information, Figure S4. The enzyme exhibited a broad lytic spectrum, lysing 25 (56%) of the 45 tested bacterial strains, comprising 43 strains from the *Bacillus* genus and two species from the genus *Priestia* (*P.flexa* and *P.megaterium*, previously classified under *Bacillus*) (Supplementary Information, Table S1). All lysed strains belonged to the *B. cereus* sensu lato group, with the exception of one *P.flexa* strain.

### 2.8. Lytic Activity at Different pH Values and Thermal Stability of Endolysin

**Figure 12:**
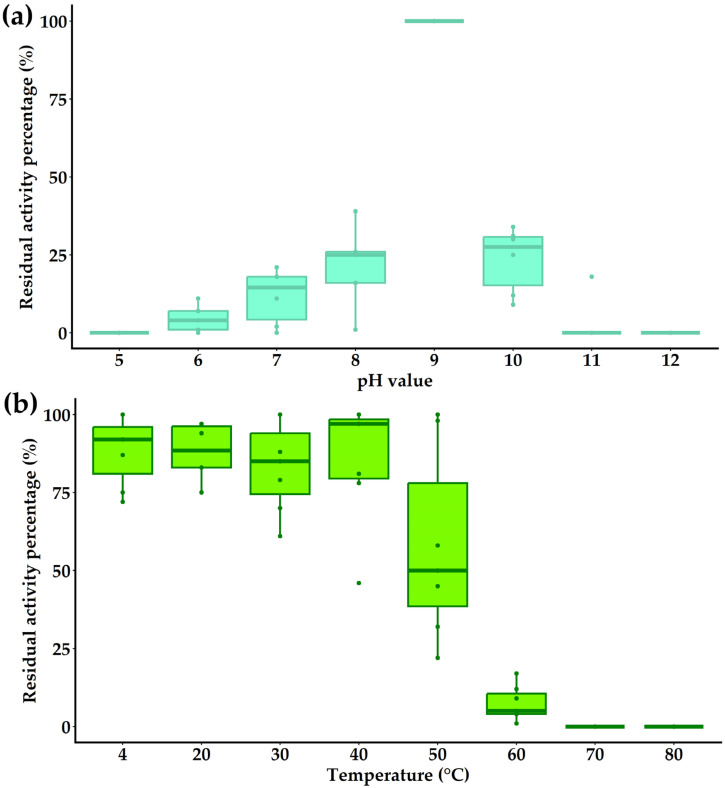
(**a**) Effect of pH on the enzymatic activity of PlyC19. (**b**) Thermal stability of the endolysin PlyC19. The figure was generated using R Studio version 4.4.3.

The enzymatic activity of the endolysin PlyC19 observed across a pH range of 6.0 to 10.0 (Figure 12a), reaching its maximum at pH 9.0. No lytic activity was detected against the bacterial strain *B. cereus* VKM B-682 at pH values of 2.0–5.0 or 11.0–12.0. PlyC19 retained full enzymatic activity after a 60 min incubation at temperatures ranging from 4 °C to 40 °C (Figure 12b). At 50 °C, the activity was reduced by approximately 50%. After incubation at 60 °C, the activity was lower than 10 ± 5.15%, and no activity was detected following incubation at 70 °C or 80 °C.

### 2.9. Ionic Strength Effect on the Bacteriolytic Activity of Endolysin

**Figure 13:**
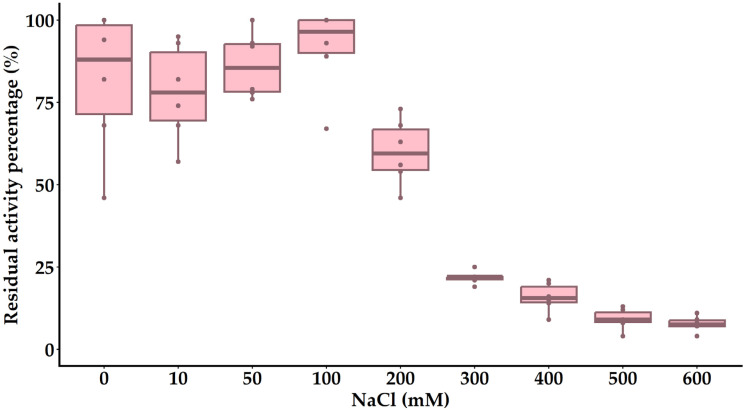
Effect of ionic strength on the bacteriolytic activity of PlyC19. The figure was generated using R Studio version 4.4.3.

The bacteriolytic activity of the endolysin PlyC19 from *Bacillus* phages B450T and B450C was maximized at a NaCl concentration of 100 mM (Figure 13). The enzyme activity decreased by 37 ± 4.5%, 75 ± 3.6%, and 89 ± 3.2% upon addition of 200 mM, 300 mM, and 600 mM NaCl, respectively. Higher NaCl concentrations adversely affected the test strain *B. cereus* VKM B-682, precluding reliable assessment of enzyme activity.

## 3. Discussion

The isolation of *Bacillus* phages B450T and B450C from a lysogenic *B. thuringiensis* strain VKM B-450 through mitomycin C induction adds to the growing knowledge base of temperate phages targeting the *B. cereus* group. This group consists of closely related species that include opportunistic pathogens responsible for foodborne illnesses, emetic syndromes, and even anthrax-like infections in immunocompromised individuals [36]. Members of *B. cereus* sensu lato pose significant public health challenges due to their spore-forming nature, environmental persistence, and antibiotic resistance, making phage-based interventions particularly appealing [36]. The investigated *Bacillus* phages B450T and B450C exhibit a siphovirus morphotype, characterized by an elongated capsid and a long, non-contractile tail, which is typical for *Bacillus*-infecting phages, as over 55% of such phages are siphoviruses [37]. This morphology optimizes attachment to the thick peptidoglycan layer of Gram-positive hosts and facilitates efficient DNA ejection across the cell wall.

Phages B450T and B450C possess a compact 41,205 bp linear double-stranded DNA chromosome with a GC content of 34.9%, which is closely aligned with the typical range of 35–45% observed in their *B. cereus* sensu lato hosts, such as *B. cereus* and *B. thuringiensis.* This near-identical GC content suggests a co-evolutionary adaptation to the host’s replication machinery, potentially facilitating efficient prophage integration and replication, though the slightly lower value may indicate a selective pressure to minimize energy costs for nucleotide synthesis in nutrient-limited environments [38]. The near-identical genomes of *Bacillus* phages B450T and B450C, differing only in two amino acid residues (Asp9Val and Leu10Ile) in the HTH_Xre domain of the repressor protein (ORF 29), illustrate how subtle genetic variations can profoundly impact phage behavior. In B450C, these changes likely disrupt DNA-binding affinity, biasing toward a lytic cycle and mimicking virulent phages, similar to mutations in the lambda CI repressor that abolish lysogeny by preventing operator occupancy [39,40,41]. This phenotypic shift—turbid plaques and incomplete lysis in B450T versus clear plaques and full inhibition in B450C—highlights the fragility of the lysis–lysogeny switch, a key regulatory hub in temperate phages that responds to host stress signals like DNA damage [42]. Such switches have been observed in various phages where mutations alter lifecycle preferences. For instance, in the temperate phage phiH5, treatment with sodium pyrophosphate led to a mutant form, phiIPLA88, that formed clear plaques due to a point mutation in the *cI* repressor gene, resulting in the loss of the start codon and rendering the phage obligately lytic [43,44]. Similarly, the temperate phage phiA72 mutated to phiIPLA35, exhibiting clear plaques after a mutation deleted 25 amino acids from the C-terminus of the repressor protein, disrupting lysogeny [43,44]. Changes in genes such as integrase and others have been reported to induce clear plaque phenotypes in temperate phages, as observed in phage ΔLCRA500, where deletions in the integrase, *gp32*, and *gp33* genes disrupt prophage establishment, preventing integration into the host genome and resulting in an obligate lytic cycle [45]. Mutations in phage-encoded tRNA genes can also disrupt lysogeny in phages that rely on tRNA-dependent integration mechanisms [46]. Additionally, environmental factors, including chelating agents (e.g., ethylenediaminetetraacetic acid (EDTA) or sodium citrate) or elevated temperatures, can promote the formation of clear-plaque mutants, as documented for phages phiH5 and lambda, where such conditions favor lytic variants by destabilizing repressor function [43,47]. These examples demonstrate how genetic or environmental perturbations can shift the balance toward the lytic development of phages.

An intriguing feature of the genomes of *Bacillus* phages B450T and B450C is the type II HicAB toxin-antitoxin (TA) system, encoded on the negative strand with the *hicA*-like toxin gene preceding the *hicB* antitoxin gene. While TA systems are ubiquitous in bacterial chromosomes and plasmids, their presence in phages is less frequent but increasingly documented [48]. In bacteria, HicAB promotes plasmid stability by post-segregational killing, induces persistence during stress by halting translation, regulates population density to prevent overcrowding, and defends against phages through abortive infection, where toxin activation kills the host to halt viral spread [49,50,51]. In phages, toxin-antitoxin (TA) modules may play similar roles: stabilizing the prophage by linking host survival to the presence of the phage, and potentially providing lysogenized hosts with competitive advantages, such as enhanced stress resistance and other benefits [51]. For example, the HicAB system was identified in a prophage of *Paracoccus* spp., and its functionality as a plasmid stabilization system was confirmed [52]. The BpsHicAB system, found in *Burkholderia pseudomallei*, contributes to the formation of persister cells [53]. In the B450T and B450C phages, the HicAB system likely promotes prophage stability in fluctuating environmental conditions and supports host survival. Currently, the role of these systems in phages is poorly understood. Future experiments investigating HicAB in phages could potentially elucidate the mechanisms by which phages ensure sustained coexistence with their bacterial hosts.

The headful packaging mechanism with site-specific initiation in *Bacillus* phages B450T and B450C, similar to that observed in SPP1-, P22-, and P1-like phages, results in circularly permuted genomes with terminal redundancy [54]. The stability of phages B450T and B450C across a pH range of 5–11 and at temperatures up to 50 °C indicates their adaptability to diverse environments, which is a desirable characteristic for therapeutic bacteriophages. However, their temperate nature raises therapeutic concerns, as they can spread genetic features in bacterial populations via horizontal gene transfer, including genes for virulence, potentially accelerating pathogen evolution [19]. The host range of phages B450T and B450C was found to be 17 (45.9%) out of the 37 *B. cereus* sensu lato strains tested. The host range is determined by adsorption specificity, which is facilitated by tail fibers that recognize polysaccharides or proteins of the bacterial cell wall [55].

PlyC19, however, circumvents phage limitations, lysing 25 (67.6%) out of the 37 *B. cereus* sensu lato strains and also *P. flexa* strain, demonstrating endolysins’ superior breadth by directly targeting conserved peptidoglycan motifs without receptor dependence [56]. The expanded host range of PlyC19, covering eight additional strains, indicates its ability to target cell components inaccessible to phages, making it potentially suitable, for example, for disrupting biofilms in infections caused by *B. cereus* group strains. The modular structure of PlyC19, featuring an Amidase_2 EAD paired with two SH3 CBDs, enables precise binding and hydrolysis, a characteristic feature of endolysins targeting Gram-positive bacteria such as *Bacillus* [57]. The low similarity of PlyC19 to known endolysins suggests evolutionary novelty, likely optimized for targeting peptidoglycan variations specific to *B. cereus* group strains.

PlyC19 exhibits an optimal bacteriolytic activity at pH of 9.0 (with a functional range of 6–10) and tolerates salt concentrations up to 100 mM NaCl, with a significant decrease in activity above 200 mM. This alkaline preference contrasts with the broader pH stability of phages B450T and B450C but aligns with characteristics typical of amidases. PlyC19 retains its full activity at up to 40 °C, but only 50% at 50 °C. This aligns with the thermal limits of phages B450T and B450C, indicating shared environmental constraints. However, PlyC19 is less heat-resistant than thermostable endolysins, such as endolysin from *Bacillus*-phage PW2 [32]. These characteristics make the endolysin PlyC19 from phages B450T and B450C suitable for applications in food safety or wound treatment, where mild environmental conditions are typically present [58]. Modifying PlyC19, such as fusing it with various CBDs, could enhance its binding affinity or stability and expand its natural bactericidal spectrum, thus widening its potential applications [31,59]. As a targeted enzybiotic against multidrug-resistant strains of the *B. cereus* group, PlyC19 demonstrates potential, comparable to other endolysins [31].

Following the ICTV standards, we propose the designation *Bquatquinnuvirus eskimopiis* for the novel phage species described in this study, with B450T and B450C being the distinct strains of the species [60]. The genus name *Bquatquinnuvirus* is derived from the B450 bacterial strain from which the prophage was isolated, incorporating Latin numerals (quattuor for 4, quinque for 5, nulla for 0), while the species name *eskimopiis* reflects the virion morphology, reminiscent of an “Eskimo Pie” ice cream.

## 4. Materials and Methods

### 4.1. Bacterial Strains and Growth Conditions

A total of 45 bacterial strains, primarily from the *Bacillus* genus and including species such as *Priestia flexa* and *Priestia megaterium* (previously classified under *Bacillus*), were used to investigate the novel phages B450T and B450C and to evaluate the lytic activity of their endolysin. These strains are detailed in Supplementary Information, Table S1. For endolysin protein expression, *Escherichia coli* strain Rosetta-gami 2(DE3) was employed as the producer strain.

Bacterial cultures were grown in Luria–Bertani (LB) medium [10 g/L tryptone, 5 g/L yeast extract, 10 g/L NaCl] or on LB agar plates (1.5% agar for single-layer plates or bilayer plates with 1.5% bottom agar and 0.5% or 0.75% top agar), supplemented with 10 mM CaCl_2_ and 10 mM MgCl_2_ to support phage infection. Double-layer agar plates were used immediately after pouring the top agar layer: no pre-incubation or drying step was applied. Cultures were incubated at 30 °C or 37 °C, depending on the experimental requirements. For the preparation of competent cells, SOB medium [20 g/L tryptone, 5 g/L yeast extract, 0.5 g/L NaCl, 0.186 g/L KCl, 0.95 g/L MgCl_2_] was used [61]. All chemical reagents used in the experiments throughout this study are detailed in Supplementary Information, Table S6.

### 4.2. Phage Isolation, Purification and Propagation

*Bacillus* phages B450T and B450C were isolated from the lysogenic bacterial strain *Bacillus thuringiensis* VKM B-450 through mitomycin C induction. It is worth emphasizing that no spontaneous phage release was ever detected from strain VKM B-450 in the absence of inducer. The two variants B450T and B450C were obtained by single-plaque isolation from the same mitomycin C-induced lysate; the clear-plaque variant B450C most likely arose as a spontaneous repressor mutant during the first propagation cycles after induction. The strain was cultured overnight in 4 mL LB medium supplemented with 10 mM CaCl_2_ and 10 mM MgCl_2_ at 30 °C. The overnight culture was diluted 1:100 in fresh LB medium with 10 mM CaCl_2_ and 10 mM MgCl_2_, and 500 µL aliquots were dispensed into a 48-well plate. The plate was incubated at 30 °C with orbital shaking in a plate reader until the culture reached an optical density at 590 nm (OD590) of approximately 0.4. Mitomycin C (final concentration 0.5 mg/mL) was added to wells containing the culture, with additional wells containing LB without culture as a control. As an internal control, wells with culture received an equivalent volume of MgSO_4_ solution. Incubation continued under the same conditions until a visible decrease in OD590 was observed in mitomycin C-treated wells compared to the internal controls. Mitomycin C-treated samples were collected in 1.5 mL Eppendorf tubes, centrifuged at 3000× *g* for 12 min at 4 °C, and the supernatant was transferred to new tubes with 10 µL chloroform added. Mitomycin C lysates were stored at 4 °C for no longer than 5 days.

The mitomycin C lysates were screened against a collection of *Bacillus* strains (Supplementary Table S1) using a spot test assay. Bacterial lawns were prepared by adding 3 mL of 0.75% LB top agar (containing 10 mM CaCl_2_ and 10 mM MgCl_2_) with 30–50 µL of a 10-fold concentrated bacterial culture (optical density at 595 nm (OD590) ≈ 1–1.2) onto a 1.5% LB bottom agar plate (with 10 mM CaCl_2_ and 10 mM MgCl_2_). Plates were dried at 25 °C for 30 min, and 10 µL of mitomycin C lysate was spotted onto the lawn, followed by overnight incubation at 30 °C. Positive results were defined by the presence of clear or turbid zones or individual plaques compared to the control (LB with mitomycin C, to exclude mitomycin C effects on the lawn).

For phage propagation, 50 µL of mitomycin C lysate was plated using the double-layer agar method on a lawn of the sensitive strain *B. thuringiensis* VKM B-446, which showed the most pronounced effect (individual plaques) in the spot test. Individual plaques were transferred to 1.5 mL tubes containing 750 µL SM+ buffer [50 mM Tris-HCl pH 7.5, 100 mM NaCl, 1 mM MgSO_4_, 0.01% gelatin, 10 mM CaCl_2_, 10 mM MgCl_2_] and 75 µL chloroform, followed by overnight incubation at 4 °C with shaking for phage extraction. This propagation-extraction cycle was repeated three times to eliminate contamination by other phages, as described previously [30]. Phage propagation and polyethylene glycol (PEG) 8000 precipitation were performed as previously reported [30,62]. The resulting high-titer phage samples were filtered through a 0.22 µm sterile filter and stored at 4 °C.

### 4.3. Transmission Electron Microscopy

Transmission electron microscopy (TEM) analysis was performed according to established protocol described in previous studies [63,64]. The morphological dimensions of phage particles were determined by analyzing transmission electron micrographs using ImageJ software version 1.53e [65]. Measurements were performed on ten individual virions, with size calibration based on the microscope-generated scale bar.

### 4.4. Host Range of Bacteriophages

Host range specificity was assessed using both the spot-test method and double-layer agar technique as previously described [30,64]. Testing was performed on a panel of 45 *Bacillus* strains, including 37 strains of the *Bacillus cereus* group along and eight additional other strains representing other *Bacillus* and *Priestia* species: *P. flexa*, *Bacillus licheniformis*, *P. megaterium*, and *Bacillus subtilis*. A detailed list of the tested strains is provided in Supplementary Information, Table S1.

### 4.5. Killing Assay of Bacteriophages

The killing assay for *Bacillus* phages B450T and B450C was conducted as previously described [30]. Bacterial cultures of *B. thuringiensis* VKM B-446 were infected with phages at multiplicities of infection (MOI) of 0.1, 1, and 10 in LB medium supplemented with 10 mM CaCl_2_ and 10 mM MgCl_2_ at 30 °C. Bacterial growth inhibition was monitored by measuring OD595 using a plate reader. Experiments were performed in triplicate, and data were analyzed using GraphPad Prism 8.4.3 to assess the lytic efficacy of the phages under the tested conditions.

### 4.6. Thermal and pH Stability Assay of Bacteriophages

The thermal and pH stability of *Bacillus* phages B450T and B450C was evaluated as described previously [30]. Phage samples were exposed to a range of temperatures (4, 20, 30, 40, 50, 60, 70, 80 and 90 °C) and pH values using appropriate buffer systems. Stability was assessed by determining the residual infectivity of the phages through a double-layer agar assay on *B. thuringiensis* VKM B-446 lawns, conducted in LB medium supplemented with 10 mM CaCl_2_ and 10 mM MgCl_2_ at 30 °C. Experiments were performed in five independent trials, and data were analyzed using GraphPad Prism 8.4.3 to quantify phage stability under varying thermal and pH conditions.

### 4.7. Genome Sequencing, Assembly and Sequence Analysis

Prior to DNA extraction, the bacteriophage preparations were treated with DNase I and RNase A following the manufacturer’s protocol. DNA purification was performed using the DNeasy Blood and Tissue Kit (Qiagen, Hilden, Germany) in accordance with the manufacturer’s instructions. The genomic DNA was subsequently sequenced on an Illumina MiSeq platform (Illumina Inc., San Diego, CA, USA) using the TruSeq DNA Library Preparation Kit (Illumina Inc., San Diego, CA, USA). De novo genome assembly was conducted with SPAdes v.3.12.0 [66]. Open reading frames (ORFs) were annotated using RASTtk v.2.0 [67], and putative protein functions were predicted through homology-based approaches employing BLAST (NCBI) [68] and HHpred [69] (accessed on May 2024). tRNA genes were identified using ARAGORN v1.2.40 [70]. Finally, the circular genome map was generated using BRIG v.0.95 [71]. The analyses were performed in May 2024.

### 4.8. Determination of DNA Packaging Strategy

The DNA packaging strategy of *Bacillus* phages B450T and B450C was investigated using bioinformatic analysis and restriction analysis, as described previously [30]. Bioinformatic analysis involved constructing a phylogenetic tree of large terminase subunits using the Neighbor-Joining method in MEGA X [72] with 1000 bootstrap replicates, visualized in FigTree v1.4.4 [73]. Restriction analysis was conducted both in silico using NEBcutter V2.0 and in vitro with restriction endonucleases NotI, HindIII, BamHI, BglII, and PstI.

### 4.9. Comparative Genomics

To identify related phages, a comparative genomics analysis of *Bacillus* phages B450T and B450C was performed based on genomic sequence similarity, as described previously [30]. The analysis was performed in May 2024. The analysis utilized whole-genome nucleotide sequence comparisons and proteomic alignments to assess phylogenetic relationships with known bacteriophages.

### 4.10. Phylogenetic Analysis and Domain Structure Analysis

The domain structure of the endolysin PlyC19 (protein_id XYL31778.1) was determined using InterProScan based on its complete amino acid sequence. The analysis generated a schematic representation of predicted domains, annotated according to the InterPro database (https://www.ebi.ac.uk/interpro/; accessed on June 2024) [74], with their respective positions within the sequence. Comparative amino acid analysis of the predicted domains was performed using BLASTp, with the “Organism” parameter set to “Viruses” and default settings for other parameters.

For phylogenetic analysis, the enzymatically active domain (EAD) of PlyC19 was compared with 35 Amidase_2 EAD sequences from other *Bacillus* phage endolysins, retrieved from the NCBI database [68] and identified using InterProScan (Supplementary Information, Table S5). Sequence alignment of the EADs was conducted in MEGA X v.10.2.6 using ClustalW [72]. Phylogenetic relationships were inferred using the maximum likelihood method based on the Jones-Taylor-Thornton model [75] in MEGA X v.10.2.6. A bootstrap consensus tree was constructed from 500 replicates [76] to visualize the evolutionary relationships among the proteins and was displayed using iTOL v.6.9.1 [77].

### 4.11. Endolysin Genes Cloning and Enzymes Purification

The nucleotide sequences of endolysin genes from *Bacillus* phages B450T and B450C genomes have 100.00% identity. The expression vector for the endolysin gene *plyC19* was constructed using the T5 exonuclease DNA assembly (TEDA) method [78]. The *plyC19* gene was amplified via PCR using high-fidelity Q5 DNA polymerase (New England Biolabs, Ipswich, MA, USA) with specific primers: forward primer Fd_19_amidase (5′-AACTTTAAGAAGGAGATATACTTATGGAAATTAGAAAAAATTTAGTTGACCC-3′) and reverse primer Rv_19_amidase (5′-AGTGGTGGTGGTGGTGGTGCGATCCGCTACCGGATTTTTCAAACTTCACATATTCACCTGA-3′). The PCR products were cloned into the pET33 expression vector between the NcoI and XhoI sites (New England Biolabs, Ipswich, MA, USA), incorporating a C-terminal 6xHis tag sequence.

Competent *E. coli* Rosetta-gami 2(DE3) cells were transformed with the resulting plasmid DNA for subsequent protein expression and purification. An overnight bacterial culture was diluted 1:100 in 250 mL LB medium supplemented with 0.1 mg/mL kanamycin (Kraspharma, Krasnoyarsk, Russia) and grown at 37 °C with shaking until reaching an OD590 of 0.4–0.8. Protein expression was induced with 0.5 mM isopropyl-β-D-1-thiogalactopyranoside (SibEnzyme, Novosibirsk, Russia), followed by incubation at 25 °C with shaking at 180 rpm for 12 h. Cells were harvested by centrifugation at 3400× *g* for 30 min at 10 °C.

All subsequent steps were performed on ice. Harvested cells were resuspended in 50 mL Buffer B containing 5 mM imidazole [40 mM Tris-HCl (Dia-M, Moscow, Russia), pH 8.0, 0.5 M NaCl, 5% glycerol (Component-Reactive, Moscow, Russia), imidazole (Dia-M, Moscow, Russia)]. Lysozyme (Reakhim, Moscow, Russia) was added to a final concentration of 0.1 mg/mL. The cell suspension was disrupted using a Qsonica Q700 ultrasonic disintegrator (Qsonica LLC, Newtown, CT, USA) with three cycles of 30 s of sonication followed by 5 min of cooling on ice. The lysate was centrifuged at 12,000× *g* for 45 min at 4 °C, and the supernatant was filtered through a 0.45 µm filter (Millipore, Billerica, MA, USA). The filtered lysate was applied to a pre-equilibrated gravity column (Smart-Lifesciences, Changzhou, China) packed with Ni-NTA-Beads-6FF resin (Smart-Lifesciences, Changzhou, China). The column was washed with 5 mL Buffer B containing 20 mM imidazole, followed by elution with 4 mL Buffer B containing 250 mM imidazole to collect the purified target protein. To remove imidazole, the eluate was dialyzed three times against storage buffer [HEPES (Dia-M, Moscow, Russia), pH 7.5, 0.5 M NaCl, 50% glycerol, 10 mM dithiothreitol (Dia-M; Moscow, Russia)] at 4 °C for 8 h. Protein concentration was determined by measuring absorbance at 280 nm (extinction coefficient = 1) using a NanoPhotometer Pearl P-360 (Implen GmbH, Munich, Germany).

Protein fractions from each purification step were analyzed by sodium dodecyl sulfate-polyacrylamide gel electrophoresis (SDS-PAGE) using a ServiceBio VII protein marker (ServiceBio, Wuhan, China). Purified endolysin aliquots were stored at −20 °C.

### 4.12. Lytic Activity Spectrum of Endolysin

The lytic activity spectrum of the endolysin PlyC19 from *Bacillus* phages B450T and B450C was assessed using a spot test assay. A 3 µL aliquot of endolysin (5.9 mg/mL) was applied to Petri dishes containing double-layered LB agar: a 1.5% LB agar bottom layer and a 0.5% LB agar top layer inoculated with a bacterial culture grown to an OD590 of approximately 0.5. The enzyme storage buffer was applied as a negative control. Plates were incubated at 25 °C overnight. Bacterial strains exhibiting a zone of lysis after incubation were classified as sensitive to the endolysin. The lytic activity of the endolysin against tested bacterial strains is detailed in Supplementary Information, Table S1.

### 4.13. Endolysin Activity Turbidimetry Assays

The enzymatic activity of the endolysin PlyC19 from *Bacillus* phages B450T and B450C under various conditions was determined using a turbidimetric assay based on the reduction in bacterial culture turbidity, as described previously [63,79] with some modifications. The test strain, *B. cereus* VKM B-682, was selected due to its high susceptibility to the lytic action of the endolysin, as demonstrated in experiments assessing the lytic activity spectrum, despite not being within the host range of phages B450T and B450C (Supplementary Information, Table S1). The strain was grown to an OD590 of approximately 0.5. In 96-well plates, 100 µL of bacterial culture was mixed with 100 µL of buffer containing the endolysin (specific buffers used are detailed for each experiment below: “pH-optimum of Bacteriolytic Activity and Thermal Stability of Endolysin” section and “Effect of NaCl on Bacteriolytic Activity of Endolysin”). The enzyme storage buffer served as a negative control. Absorbance measurements were performed using a FilterMax F5 plate reader (Molecular Devices, San Jose, CA, USA) at 595 nm, recorded every minute for 2 h at 37 °C with vigorous shaking. Experiments were conducted with at least three independent trials. Relative endolysin activity was expressed as a percentage, with 100% representing the maximum activity observed in each replicate. Data analysis for relative activity was performed using Microsoft Excel 2016.

### 4.14. pH-Optimum of Bacteriolytic Activity and Thermal Stability of Endolysin

The effect of pH on the bacteriolytic activity of the endolysin PlyC19 was evaluated using the same buffer systems employed for phage stability assays (see section “Thermal and pH Stability Assay of bacteriophages”). Activity was measured as described in the “Endolysin Activity Turbidimetry Assays” section, with a final endolysin concentration of 2.5 µM.

To assess thermal stability of endolysin, the endolysin was mixed with 0.2 M glycine-NaOH buffer (pH 9.0) to a final concentration of 10 µM and incubated at temperatures of 4, 10, 20, 30, 40, 50, 60, 70, and 80 °C for 1 h. After cooling to room temperature, the thermally treated samples were analyzed for residual bacteriolytic activity using the “Endolysin Activity Turbidimetry Assays” protocol.

### 4.15. Effect of NaCl on Bacteriolytic Activity of Endolysin

The influence of NaCl concentration on the bacteriolytic activity of the endolysin PlyC19 from *Bacillus* phages B450T and B450C was evaluated using the protocol described in the “Endolysin Activity Turbidimetry Assays” section. NaCl concentrations tested were 10 mM, 50 mM, 100 mM, and increasing increments of 100 mM up to 800 mM. The final endolysin concentration was 2.5 µM, and assays were conducted in 0.2 M glycine-NaOH buffer (pH 9.0). Experiments were performed in at least three independent trials, with relative activity calculated as outlined in the “Endolysin Activity Turbidimetry Assays” section.

### 4.16. Statistical Analysis

Statistical analysis for experiments involving *Bacillus* phages B450T and B450C was conducted using GraphPad Prism 8.4.3 [80,81] as described in our previous publications [30,63]. A *p*-value of ≤0.05 was considered statistically significant. One-way ANOVA with repeated measures was used to compare phage concentrations in thermal/pH stability tests, and control vs. test samples in lytic activity spectrum experiments. Two-way ANOVA with repeated measures analyzed growth curve differences in killing assays (different MOIs). Statistical analysis of endolysin activity experiments and graph generation were performed using R Studio version 4.4.3 [82].

### 4.17. Accession Number

The genomic sequences of phages 450T and 450C are available in GenBank (accession PV695473 and PV695472, respectively). The project, entitled “Prophages of *Bacillus cereus* sensu lato”, is registered under BioProject accession number PRJNA861678. The BioSample accession numbers for 450T and 450C are SAMN48738070 and SAMN48738069, respectively. The raw sequencing reads are deposited in the SRA (under accession numbers: SRR33743889 for 450T and SRR33743888 for 450C).

## 5. Conclusions

The investigation of *Bacillus* phages B450T and B450C, derived from a lysogenic *B. thuringiensis* strain, advances our knowledge of temperate phages within the *B. cereus* group. These phages, exhibiting a siphovirus morphotype and a compact 41,205 bp genome, demonstrate co-evolutionary adaptations to their hosts, including a type II HicAB toxin-antitoxin system that likely enhances prophage stability and host survival in fluctuating environments. The limited host range of phages B450T and B450C underscores their ecological specificity, while their endolysin PlyC19′s broader lytic activity highlights its potential as a targeted enzybiotic against multidrug-resistant pathogens. We propose the taxonomic designation *Bquatquinnuvirus eskimopiis* for these phages, reflecting their prophage origin and morphological characteristics. This study expands bacteriophage taxonomy, deepens insights into temperate phages, and underscores the therapeutic advantages of endolysins over intact phages, laying the foundation for future research into phage-derived therapeutics. Looking ahead, continued advances in phage genome editing over the next decade will enable the targeted modification of temperate phages–converting them into strictly lytic variants, eliminating lysogenic pathways, or harnessing their unique genetic modules to enhance lytic phages–thereby unlocking the long-underestimated therapeutic potential of the temperate phage gene pool and establishing them as safe, versatile, and highly effective tools in modern phage therapy.

## Data Availability

The annotated complete genomes of *Bacillus* phages B450T and B450C are available in GenBank under accession numbers PV695473 and PV695472, respectively. The project, titled “Prophages of *Bacillus cereus* sensu lato,” is registered under BioProject accession number PRJNA861678.

## Supplementary Materials

The following supporting information can be downloaded at: https://www.mdpi.com/article/10.3390/ijms27010131/s1.
