# Supplementary material for: Novel Bacillus-Infecting Phage Bquatquinnuvirus eskimopiis (Strains B450T and B450C), Founder of a New Genus, and the Properties of Its Endolysin
Source: Int J Mol Sci. 2025 Dec 22;27(1):131. doi: 10.3390/ijms27010131 (PMC12786184; doi:10.3390/ijms27010131)
Supplement: Supplementary file 1 [file ijms-27-00131-s001.zip › ijms-3996828-supplementary.pdf]

Novel *Bacillus*-infecting phage *Bquatquinnuvirus eskimopiis* (Strains B450T and B450C), Founder of a New Genus, and The Properties of Its Endolysin

Olesya A. Kazantseva[1,*], Olga N. Koposova[1], Irina A. Shorokhova[1], Vladislav A. Kulyabin[1], Andrey M. Shadrin[1]

[1] G.K. Skryabin Institute of Biochemistry and Physiology of Microorganisms, Pushchino Scientific Center for Biological Research of the Russian Academy of Sciences, Federal Research Center, Prospect Nauki, 5, 142290 Pushchino, Russia

* Correspondence: olesyakazantseva@bk.ru (O.A.K).

Supplementary Information:

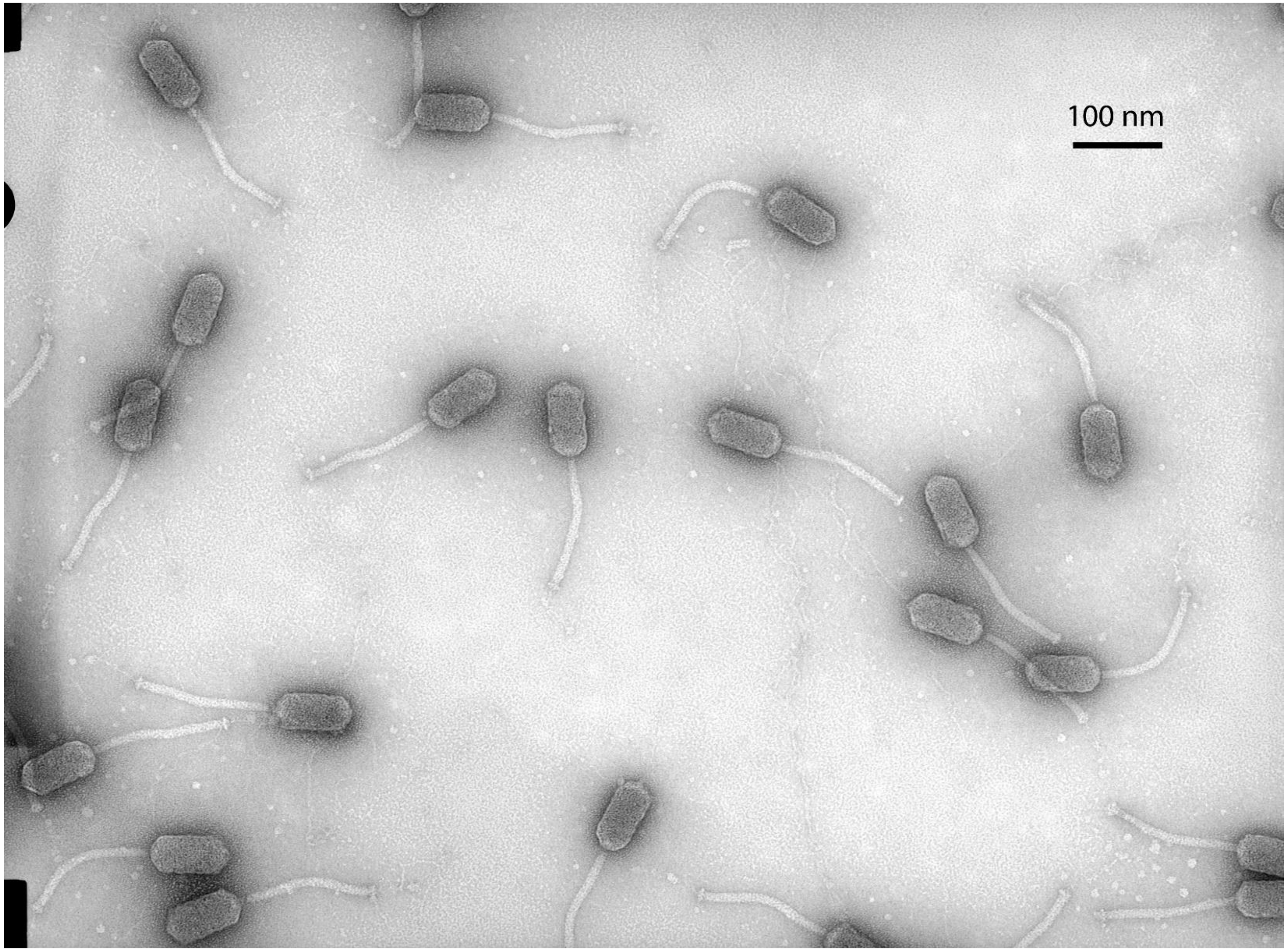

Figure S1. Transmission electron microscopy of the *Bacillus* phage B450T. This is the original TEM micrograph used to generate Figure 2 in the main text. The image was taken using a JEM 1200EX (JEOL, Japan) transmission electron microscope at 80 kV accelerating voltage on Kodak film SO-163 (Kodak, Cat. # 74144, Hatfield, PA, USA).

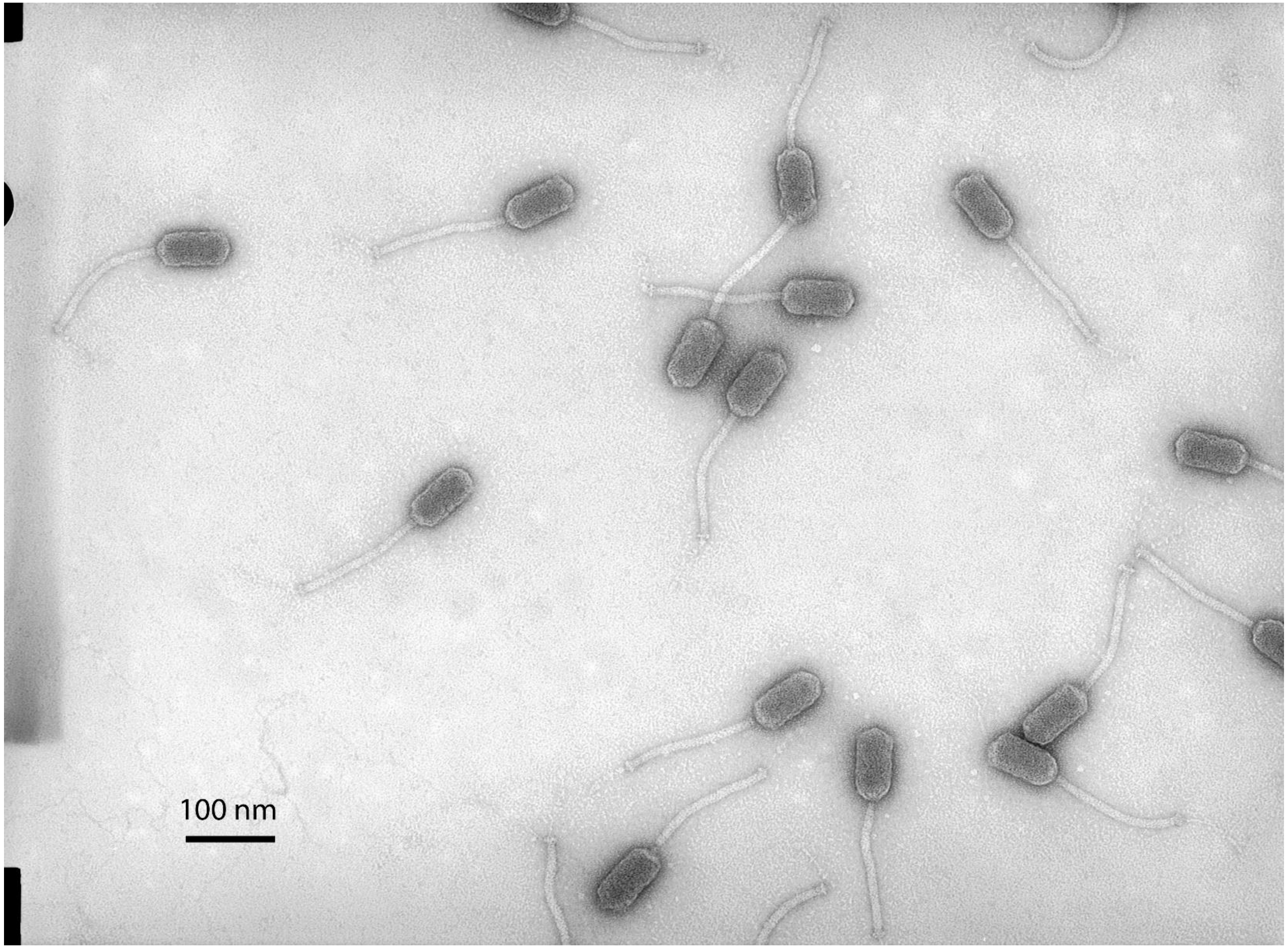

Figure S2. Transmission electron microscopy of the *Bacillus* phage B450C. This is the original TEM micrograph used to generate Figure 2 in the main text. The image was taken using a JEM 1200EX (JEOL, Japan) transmission electron microscope at 80 kV accelerating voltage on Kodak film SO-163 (Kodak, Cat. # 74144, Hatfield, PA, USA).

Table S1. The host range of phages B450T and B450C determined on 45 *Bacillus* strains.

№	Bacterial species	Strain	Source	Phage lysis		Endolysin lysis
				B450T	B450C	PlyC19
1	*B. cereus*	VKM B-13	VKM	+	+	+
2	*B. cereus*	VKM B-15	VKM	+	+	-
3	*B. cereus*	VKM B-370	VKM	+	+	-
4	*B. cereus*	VKM B-373	VKM	+	+	+
5	*B. cereus*	VKM B-374	VKM	+	+	-
6	*B. cereus*	VKM B-383	VKM	+	+	+
7	*B. cereus*	VKM B-445	VKM	+	+	-
8	*B. cereus*	VKM B-473	VKM	+	+	+
9	*B. cereus*	VKM B-491	VKM	-	-	+
10	*B. cereus*	VKM B-504^T	VKM	-	-	+
11	*B. cereus*	VKM B-681	VKM	-	-	-
12	*B. cereus*	VKM B-682	VKM	-	-	+
13	*B. cereus*	VKM B-683	VKM	+	+	+
14	*B. cereus*	VKM B-684	VKM	-	-	-
15	*B. cereus*	VKM B-686	VKM	-	-	-
16	*B. cereus*	VKM B-687	VKM	-	-	-
17	*B. cereus*	VKM B-688	VKM	+	+	+
18	*B. cereus*	VKM B-771	VKM	-	-	-
19	*B. cereus*	VKM B-810	VKM	-	-	+
20	*B. cereus*	VKM B-811	VKM	-	-	+
21	*B. cereus*	VKM B-812	VKM	-	-	-
22	**P. flexa*	AVS-02	Laboratory collection	-	-	+
23	*B. licheniformis*	B-433	VKM	-	-	-
24	*B. licheniformis*	B-511^T	VKM	-	-	-
25	**P. megaterium*	MS941	MoBiTec	-	-	-
26	*B. pumilus*	AVS-01	Laboratory collection	-	-	-
27	*B. subtilis*	B-501^T	VKM	-	-	-
28	*B. subtilis*	168 His III	Laboratory collection	-	-	-
29	*B. subtilis*	WB 800n	MoBiTec	-	-	-
30	*B. tropicus*	ATCC 4342	ATCC	-	-	+
31	*B. thuringiensis*	VKM B-83	VKM	-	-	+
32	*B. thuringiensis*	VKM B-84	VKM	-	-	+
33	*B. thuringiensis*	VKM B-85	VKM	-	-	+
34	*B. thuringiensis*	VKM B-440	VKM	+	+	+
35	*B. thuringiensis*	VKM B-443	VKM	-	-	+
36	*B. thuringiensis*	VKM B-446	VKM	+	+	+
37	*B. thuringiensis*	VKM B-447	VKM	-	-	+
38	*B. thuringiensis*	VKM B-450	VKM	-	-	+
39	*B. thuringiensis*	VKM B-453	VKM	+	+	+
40	*B. thuringiensis*	VKM B-454	VKM	+	+	+
41	*B. thuringiensis*	VKM B-1555	VKM	+	+	+
42	*B. thuringiensis*	VKM B-1557	VKM	+	+	+
43	*B. thuringiensis*	ATCC 35646	ATCC	+	+	-
44	*B. toyonensis*	IP 5832	probiotic"Bactisubtil"[1]	-	-	-
45	*B. mycoides*	KBAB4	[2]	-	-	-

Source abbreviations: VKM: All-Russian Collection of Microorganisms; ATCC: American Type Culture Collection. * *P. flexa* and *P. megaterium* are species of the genus *Priestia*, previously classified under the genus *Bacillus.*

Table S2. Annotation of *Bacillus* phages B450T and B450C.

ORF№	Start codon	Stop codon	Strand	Blast results		Conserved domains, Blast		Hhpred results	Annotation
				Name	E-val	Name, (region)	E-val	(Prob./E-val)	
1	1	732	+	putative terminase small subunit [*AnoxyBacillus* phage A403]<br><br>phage-related terminase small subunit-like protein [*Bacillus* virus 250]<br><br>TPA: MAG TPA: Small Terminase [*Podoviridae* sp.]	3.0e-76<br><br>9.0e-76<br><br>2.0e-62	YjcR (1-241)<br><br>Sigma70_r4_2 (1-26)	1.43e-50<br><br>2.43e-04	COG5484; YjcR; Uncharacterized protein YjcR, contains N-terminal HTH domain [Function unknown] (99.85/6.60e-20)<br><br>P51718; VPP_BPHC1 Probable terminase, ATPase subunit, Haemophilus phage HP1 (strain HP1c1) (95.82/1.10e-02)<br><br>d1ijwc_; a.4.1.2 (C:) HIN recombinase (DNA-binding domain) (95.8/1.20e-02)<br><br>7LW0_A Terminase small subunit; DNA Packaging, Terminase,	putative terminase, small subunit

								VIRAL PROTEIN; 2.32A {*Escherichia* phage lambda} (95.17/ 0.055)	
2	729	1949	+	TPA: MAG TPA: large terminase [*Podoviridae* sp.]<br>TPA: MAG TPA: large terminase [*Podoviridae* sp.]<br>TPA: MAG TPA: large terminase [*Podoviridae* sp. ct16M3]	0.0e+00<br>0.0e+00<br>0.0e+00	phage_term_2 (26-399)<br>Terminase_6 (87-207)	2.00e-45<br>2.56e-05	Q6QGD2; TERL_BPT5 Terminase, large subunit, *Escherichia* phage T5 (100.0/7.60e-40)<br>P54308; TERL_BPSPP Terminase, large subunit, *Bacillus* phage SPP1 (100.0/2.00e-38)<br>P16938; TERL_BPLP7 Terminase, large subunit (Fragment), *Enterobacteria* phage LP7 (100.0/3.30e-38)	terminase, large subunit
3	1962	3443	+	TPA: MAG TPA: portal protein [*Podoviridae* sp. ctTRl7]	8.0e-85	Phage_prot_Gp6 (36-456)	1.16e-64	P54309; PORTL_BPSPP Portal protein, *Bacillus* phage SPP1 (100.0/6.10e-39)<br>Q24LJ4; PORTL_BPPCD Portal protein, *Clostridium* phage	portal protein

				TPA: MAG TPA: PORTAL PROTEIN [*Myoviridae* sp.]<br><br>TPA: MAG TPA: PORTAL PROTEIN [*Myoviridae* sp.]	5.0e-83<br><br>7.0e-82			phiCD119 (strain *Clostridium* difficile/United States/Govind/2006) (100.0/1.20e-37)<br><br>O64207; PORTL_BPMD2 Portal protein, *Mycobacterium* phage D29 (100.0/4.20e-34)	
4	3562	4377	+	head morphogenesis protein [Phage Altai3]<br><br>TPA: MAG TPA: minor capsid protein [*Podoviridae* sp.]<br><br>TPA: MAG TPA: minor capsid protein [*Podoviridae* sp.]	2.0e-53<br><br>3.0e-45<br><br>4.0e-45	phageSPP1_gp7 (130-236)<br><br>COG5585 (1-270)	1.04e-25<br><br>1.57e-11	Q38442; GP7_BPSPP Minor head protein GP7, *Bacillus* phage SPP1 (99.98/2.20e-29)<br><br>COG5585; COG5585; NAD+ asparagine ADP-ribosyltransferase [Signal transduction mechanisms] (99.96/5.50e-27)<br><br>Q04765; VSP1_BPLLH Structural protein, *Lactococcus* phage LL-H (99.9/8.50e-22)	minor capsid protein

5	4361	4543	+	TPA: MAG TPA: helix-turn-helix domain protein [*Podoviridae* sp.]<br><br>TPA: MAG TPA: helix-turn-helix domain protein [*Podoviridae* sp.]<br><br>TPA: MAG TPA: helix-turn-helix domain protein [*Podoviridae* sp.]	5.0e-05<br><br>2.0e-04<br><br>2.0e-04	HTH_17<br>(15-56)	1.93e-04	P25135; VG090_BPPF1 10.1 kDa protein, *Pseudomonas* phage Pf1 (99.22/1.20e-09)<br><br>COG3311; AlpA; Predicted DNA-binding transcriptional regulator AlpA [Transcription, Mobilome: prophages, transposons] (99.03/5.90e-09)<br><br>P07695; VCOX_BPP2 Regulatory protein cox, *Escherichia* phage P2 (99.08/7.50e-09)	HTH domain-containing protein
6	4629	5270	+	TPA: MAG TPA: Major head protein [*Myoviridae* sp.]<br><br>TPA: MAG TPA: Major head protein [*Myoviridae* sp.]	7.0e-17<br><br>1.0e-16	DUF4355<br>(62-173)	1.92e-08	6B0X_g Scaffold protein; major capsid protein, HK97-like fold, scaffolding protein, procapsid, VIRUS (99.9/ 4e-22)<br><br>Q05222; SCAF_BPML5 Probable capsid assembly scaffolding	major capsid protein

				TPA: MAG TPA: Major head protein [*Myoviridae* sp.]	1.0e-16			protein, Mycobacterium phage L5 (98.26/3.00e-05)<br><br>O64209; SCAF_BPMD2 Probable capsid assembly scaffolding protein, Mycobacterium phage D29 (98.09/5.50e-05)	
7	5285	6223	+	TPA: MAG TPA: Major capsid protein [*Podoviridae* sp.]<br><br>TPA: MAG TPA: Major capsid protein [*Podoviridae* sp.]<br><br>TPA: MAG TPA: Major capsid protein [*Podoviridae* sp.]	8.0e-68<br><br>4.0e-67<br><br>2.0e-65	DUF5309<br>(14-295)	6.07e-73	B0FIH3; CAPSD_BPE32 Major capsid protein, *Escherichia* phage Phieco32 (100.0/7.90e-38)<br><br>P85987; CAPSD_BPSK1 Major capsid protein, *Serratia* phage (100.0/8.00e-37)<br><br>P85226; VPN2_BPPHE Putative major capsid protein, *Enterococcus* phage phiEF24C (99.92/5.00e-23)	major capsid protein
8	6241	6429	+	-	-	-	-	('-', '-')	hp

9	6432	6773	+	TPA: MAG TPA: tail connector protein [*Podoviridae* sp.]<br><br>TPA: MAG TPA: portal protein, 15 protein, head protein, viral infection, tailed.2A [*Podoviridae* sp.]<br><br>putative head-tail connector [*Streptococcus* phage P738]	7.0e-17<br><br>2.0e-16<br><br>3.0e-15	Phage_connect_1 (1-84)	7.87e-12	Q38584; HCP15_BPSPP Head completion protein gp15, *Bacillus* phage SPP1 (99.7/7.20e-16)<br><br>cd08055; gp15; Head-Tail Connector Protein gp15 of Bacteriophage SPP1 and similar proteins. The bacteriophage SPP1 gp15 protein I (99.69/8.10e-16)<br><br>d1xn8a_; a.229.1.1 (A:) Hypothetical protein YqbG {*Bacillus* subtilis [TaxId: 1423]} (99.67/4.20e-15)	head completion protein
10	6770	7087	+	-	-	-	-	COG5614; COG5614; Bacteriophage head-tail adaptor [Mobilome: prophages, transposons] (98.5/4.30e-06)	head completion protein

								O48446; HCP16_BPSPP Head completion protein gp16, *Bacillus* phage SPP1 (98.39/2.20e-05)<br><br>O64214; VG20_BPMD2 Gene 20 protein, Mycobacterium phage D29 (95.78/3.00e-02)	
11	7071	7499	+	TPA: MAG TPA: type I neck protein [*Podoviridae* sp.]<br><br>TPA: MAG TPA: Minor capsid protein [*Podoviridae* sp.]<br><br>TPA: MAG TPA: type I neck protein [*Podoviridae* sp.]	3.0e-07<br><br>3.0e-07<br><br>9.0e-07	-	-	COG5005; COG5005; Mu-like prophage protein gpG [Mobilome: prophages, transposons] (99.69/4.20e-16)<br><br>Q01261; GPG_BPMU Putative capsid assembly protein G, *Escherichia* phage Mu (99.23/1.20e-10)<br><br>P03731 protein Z *Escherichia* phage lambda (95.99/ 0.067)	gpG-like tail completion protein

12	7492	7917	+	-	-	-	-	O48448; COMPL_BPSPP Tail completion protein gp17, *Bacillus* phage SPP1 (99.84/4.90e-19)<br><br>O64216; VG22_BPMD2 Gene 22 protein, *Mycobacterium* phage D29 (98.99/3.70e-08)<br><br>O64326 TTTP_BPN15 Tail tube terminator protein *Escherichia* phage N15 (98.04/ 0.00075)<br><br>P03732; TTTP_LAMBD Tail tube terminator protein, *Escherichia* phage lambda (98.07/3.60e-04)	tail completion protein
13	7919	8446	+	TPA: MAG TPA: major tail protein [*Myoviridae* sp.]	1.0e-17<br><br>6.0e-17	COG5437<br><br>(19-160)	2.06e-12	2K4Q_A; Major tail protein V; gpV, Bacteriophage Lambda, Major tail protein, VIRAL PROTEIN; NMR {*Enterobacteria* phage lambda} (99.76/ 1.5e-16)	tail tube protein

				TPA: MAG TPA: major tail protein [*Podoviridae* sp.]<br><br>TPA: MAG TPA: major tail protein [*Podoviridae* sp.]	7.0e-17			P85503; STRU3_BPPAJ Structural protein 3, *Pseudomonas* phage PAJU2 (99.63/5.30e-14)<br><br>A9CRB8; TAIL_BPMR1 Putative tail protein, *Staphylococcus* phage phiMR11 (99.58/1.50e-13)<br><br>6P3E_G Tail tube protein; Tail tube, siphoviridae, helical, VIRAL PROTEIN; 5.4A {*Escherichia* phage lambda} (99.58/ 2.6e-13)	
14	8449	8859	+	-	-	-	-	P79679; TUBE_BPMU Tail tube protein, *Escherichia* phage Mu (76.82/4.80e+00)	putative tail tube protein
15	8883	9158	+	-	-	-	-	P03735; GT_LAMBD Tail assembly protein GT, *Escherichia* phage lambda (97.92/ 0.00014)<br><br>O64312; TAPFS_BPP2 Tail assembly protein E, *Escherichia* phage P2 (90.36/3.30e-01)	tail assembly protein

16	9158	11884	+	tail length tape-measure protein [*Bacillus* phage vB_BtS_BMBtp15]<br><br>tail tape measure protein [*Bacillus* phage vB_BthS-HD29phi]<br><br>tail length tape-measure protein [*Bacillus* phage BMBtp1]	3.0e-145<br><br>2.0e-144<br><br>2.0e-144	YqbO (15-421)<br><br>PhageMin_Tail (105-299)<br><br>PTZ00121 (570-767)	5.20e-36<br><br>1.17e-18<br><br>7.75e-09	Q9ZXA5; TMP_BPPHC Probable tape measure protein, *Streptomyces* phage phiC31 (100.0/2.60e-28)<br><br>Q24LI1; TMP_BPPCD Probable tape measure protein, *Clostridium* phage phiCD119 (strain *Clostridium* difficile/United States/Govind (100.0/1.30e-26)<br><br>E7DNB6; TMP_BPDP1 Tape measure protein, *Pneumococcus* phage Dp-1 (100.0/6.30e-26)	tape measure protein
17	11900	13381	+	tail family protein [*Bacillus* phage phi4J1]<br><br>tail family protein [*Staphylococcus* phage SpaA1]	0.0e+00<br><br>0.0e+00	Sipho_tail (24-240)<br><br>phi3626_gp14_N (3-131)	7.77e-21<br><br>1.80e-18	5LY8_A; Tail component; bacteriophage infection, *LactoBacillus casei* (100/ 1.9e-30)<br><br>O48459; DIT_BPSPP Distal tail protein, *Bacillus* phage SPP1 (99.73/3.20e-16)	distal tail protein

				tail component [*Bacillus* phage vB_BthS-TP21T]	0.0e+00	YomH<br>(1-164)	4.18e-18	4V96_AT; ORF46; Distal tail protein, Receptor-binding protein (99.76/ 1.4e-16)<br><br>COG4722; YomH; Phage-related protein [Mobilome: prophages, transposons] (99.72/8.00e-16)<br><br>O64221; VG27_BPMD2 Minor tail protein Gp27, *Mycobacterium* phage D29 (99.38/2.20e-11)	
18	13378	18243	+	minor structural protein [*Bacillus* phage phi4J1]<br><br>baseplate hub protein and central tail fiber [*Bacillus* phage vB_BtS_B83]	0.0e+00<br><br>0.0e+00	put_anti_recept<br>(30-352)<br><br>Prophage_tail<br>(98-336)	1.34e-76<br><br>1.12e-10	Q0PDK6; FIBER_BPSPP Tail spike protein, *Bacillus* phage SPP1 (99.97/1.40e-27)<br><br>COG4926; PblB; Phage-related protein [Mobilome: prophages, transposons] (99.92/2.10e-23)	baseplate hub protein and central tail fiber

				tail fiber domain-containing protein [*Staphylococcus* phage SpaA1]	0.0e+00	Smc (346-594)	1.86e-09	6V8I_AE; Tail-Associated Lysin, gp59; phage tail, tail tip, tape measure protein, VIRAL PROTEIN (99.91/ 1.1e-21)<br><br>A8E283; VPN7_BPPHE Tail fiber protein, Enterococcus phage phiEF24C (99.8/1.00e-17)	
19	18259	18633	+	hp	hp	-	-	COG2002; AbrB; Bifunctional DNA-binding transcriptional regulator of stationary/sporulation/toxin gene expression and antitoxin c(99.2/4.90e-11)<br><br>d2fy9a1; b.129.1.3 (A:1-54) Putative transition state regulator ABH {*Bacillus subtilis*} (98.62/3.50e-07)<br><br>d1yfba_; b.129.1.3 (A:) Transcription-state regulator AbrB, the N-terminal DNA recognition	transition state regulatory protein AbrB

								domain {*Bacillus subtilis*} (98.39/2.60e-06)	
20	20006	18795	-	mobile element protein [*Streptococcus* phage Javan580]<br>mobile element protein [*Streptococcus* phage Javan579]<br>mobile element protein [*Streptococcus* phage phiZJ20091101-3]	1.0e-86<br>4.0e-86<br>5.0e-85	transpos_IS110 (32-381)<br>Transposase_20 (273-360)<br>COG3547 (228-371)	6.00e-42<br>3.17e-24<br>2.26e-06	COG3547; COG3547; Transposase [Mobilome: prophages, transposons] (100.0/1.40e-37)<br>COG1498; SIK1; RNA processing factor Prp31, contains Nop domain [Translation, ribosomal structure and biogenesis] (99.51/3.00e-12)<br>KOG2572; Ribosome biogenesis protein - Nop58p/Nop5p [RNA processing and modification, Translation, ribosomal structure and biogen (97.48/1.10e-02)	transposase
21	20212	20706	+	holin family protein [*Bacillus* phage phi4J1]<br>holin [*Bacillus* phage BVE2]	2.0e-83<br>1.0e-82	COG4824 (25-156)	3.90e-37	COG4824; COG4824; Phage-related holin (Lysis protein) [Mobilome: prophages, transposons] (99.96/2.70e-27)	holin

				holin [*Bacillus* phage vB_BtS_BMBtp13]	4.0e-82			Q9ZXD8; VLYS_BPPH1 Probable holin, *Bacillus* phage phi105 (99.96/3.30e-27)<br><br>P07539; HOLIN_BPPZA Antiholin, *Bacillus* phage PZA (99.91/3.00e-23)	
22	20696	21634	+	N-acetylmuramoyl-L-alanine amidase [*Bacillus* phage Waukesha92]<br><br>N-acetylmuramoyl-L-alanine amidase [*Bacillus* phage vB_BthS-TP21T]<br><br>N-acetylmuramoyl-L-alanine amidase [*Bacillus* phage vB_BthS-HD29phi]	0.0e+00<br><br>0.0e+00<br><br>3.0e-126	CwlA (3-181)<br><br>PGRP (22-141)<br><br>sporang_Gsm (187-309)	4.13e-55<br><br>2.52e-25<br><br>5.58e-12	d1yb0a1; d.118.1.1 (A:1-157) N-acetylmuramoyl-L-alanine amidase PlyG {*Anthrax Bacillus* (*Bacillus anthracis*)} (99.89/1.60e-21)<br><br>COG5632; CwlA; N-acetylmuramoyl-L-alanine amidase CwlA [Cell wall/membrane/envelope biogenesis] (99.81/5.30e-18)<br><br>d2cb3a1; d.118.1.1 (A:174-344) Peptidoglycan-recognition protein-LE {Fruit fly (*Drosophila*	N-acetylmuramoyl-L-alanine amidase

								melanogaster) [TaxId: 7227]} (99.73/5.20e-17)	
23	22192	21863	-	TPA: MAG TPA_asm: Protein of unknown function (DUF4064) [*Podoviridae* sp.]<br><br>DUF4064 domain-containing protein [*Bacillus* phage Waukesha92]	3.0e-07<br><br>2.0e-05	-	-	('-', '-')	hp
24	22330	22548	+	hp	hp	-	-	7P5X_AX; PafC; transcription, activator, sigma adaption, RNA polymerase, RNAP, PafB, PafC, PafBC, Mycobacterium, smegmatis (99.31/ 1.6e-10)<br><br>7TB6_A; S. maltophilia CapW; helix-turn-helix, winged helix, WYL, transcription factor, DNA binding protein (99.22/ 4.2e-10)	transcriptional regulator

								7T8L_A; BrxR; Phage restriction, BREX, DNA binding, regulatory, Acinetobacter, Bacteriophage Exclusion, DNA binding protein (99.2/ 5.1e-10)<br><br>COG2378; YafY; Predicted DNA-binding transcriptional regulator YafY, contains an HTH and WYL domains [Transcription] (99.28/2.10e-10)	
25	22563	22856	+	YolD-like family protein [*Paenibacillus* phage Diva]<br><br>YolD-like protein [*Paenibacillus* phage Fern]	1.0e-05<br><br>1.0e-05	YolD<br>(10-94)	1.18e-06	SCOP_d1sg5a1; b.137.1.2 (A:1-86) Inhibitor of Rho Rof {*Escherichia coli* [TaxId: 562]} (97.41/ 0.011)<br><br>d1sg5a1; b.137.1.2 (A:1-86) Inhibitor of Rho Rof {*Escherichia coli* [TaxId: 562]} (95.78/2.10e-01)	YolD-like family protein

				YolD-like protein [*Paenibacillus* phage Xenia]	2.0e-05				
26	23706	22873	-	helix_turn_helix protein [*Bacillus* phage BM5]<br><br>putative cytosolic protein [*Bacillus* phage vB_BspM_MarvelLand]<br><br>DNA binding protein [*Bacillus* phage Spock]	2.0e-115<br><br>3.0e-112<br><br>2.0e-95	-	-	COG5529; COG5529; Pyocin large subunit [Secondary metabolites biosynthesis, transport and catabolism] (98.48/1.30e-06)<br><br>P19654; REPL_BPP1 Replication protein repL, *Escherichia* phage P1 (98.26/6.70e-06)<br><br>P03688; VRPO_LAMBD Replication protein, *Escherichia* phage lambda (97.77/1.40e-04)	DNA binding protein
27	25417	23984	-	site-specific recombinase for integration and excision [*AnoxyBacillus* phage A403]	1.0e-137	Resolvase (3-151)<br><br>PinE	1.08e-53<br><br>2.84e-42	COG1961; PinE; Site-specific DNA recombinase related to the DNA invertase Pin [Replication, recombination and repair] (99.95/2.50e-25)	site-specific DNA recombinase

				putative serine integrase [*VirgiBacillus* phage Mimir87]<br><br>recombinase [Deep-sea *thermophilic* phage D6E]	5.0e-131<br><br>1.0e-127	(1-219)<br><br>Recombinase<br>(173-263)	4.53e-19	Q38199; GIN_BPD10 Serine recombinase gin, *Escherichia* phage D108 (99.81/9.10e-18)<br><br>P21021; F16_VACCC Protein F16 *Vaccinia* virus (strain Copenhagen) (99.75/1.20e-17)	
28	25883	25449	-	TPA: MAG TPA: IrrE protein [*Podoviridae* sp.]<br><br>TPA: MAG TPA: IrrE protein [*Myoviridae* sp.]<br><br>TPA: MAG TPA: IrrE protein [*Podoviridae* sp.]	1.0e-33<br><br>2.0e-32<br><br>1.0e-31	ImmA<br>(14-142)<br><br>Peptidase_M78<br>(25-113)	3.83e-22<br><br>8.14e-19	P10426; YIM2_BPPH1 Uncharacterized immunity region protein, *Bacillus* phage phi105 (99.93/1.40e-23)<br><br>COG2856; ImmA; Zn-dependent peptidase ImmA, M78 family [Posttranslational modification, protein turnover, chaperones] (99.88/2.50e-21)<br><br>COG3800; COG3800; Predicted transcriptional regulator [General	ImmA/IrrE family metallo-endopeptidase domain-containing protein

								function prediction only] (99.84/1.00e-19)	
29	26313	25906	-	TPA: MAG TPA: repressor protein [*Podoviridae* sp.]<br><br>TPA: MAG TPA_asm: repressor protein [*Podoviridae* sp.]<br><br>TPA: MAG TPA: repressor protein [*Podoviridae* sp.]	2.0e-37<br><br>2.0e-34<br><br>2.0e-32	HTH_XRE (5-59)	2.26e-05	P06153; RPC_BPPH1 Immunity repressor protein, *Bacillus* phage phi105 (99.01/5.10e-07)<br><br>P04132; RPC_BPP2 Repressor protein C, *Escherichia* phage P2 (98.28/1.30e-05)<br><br>COG5606; COG5606; Predicted DNA-binding protein, XRE-type HTH domain [General function prediction only] (97.97/7.50e-05)	transcriptional regulator (repressor protein)
30	26595	26795	+	helix-turn-helix transcriptional regulator [*Staphylococcus* virus phiETA2]	1.0e-13	HTH_XRE (9-62)	2.10e-06	P04132; RPC_BPP2 Repressor protein C, *Escherichia* phage P2 (98.92/6.00e-08)<br><br>P13772; IMMF_BPPH1 ImmF control region 10 kDa protein,	transcriptional regulator (repressor protein)

				helix-turn-helix transcriptional regulator [*Staphylococcus* virus 187]<br><br>helix-turn-helix transcriptional regulator [*Paenibacillus* phage HB10c2]	1.0e-13<br><br>4.0e-13			*Bacillus* phage phi105 (98.59/1.30e-06)<br><br>d2icta_; a.35.1.3 (A:) Antitoxin HigA {*Escherichia coli* [TaxId: 562]} (98.54/2.10e-06)	
31	26792	26938	+	-	-	-	-	('-', '-')	hp
32	26965	27120	+	putative NHN endonuclease [*Bacillus* phage vB_*Bacillus*_1020A]<br><br>gp68 [*Listeria* phage B054]<br><br>TPA: MAG TPA: Metallo-beta-lactamase	5.0e-12<br><br>2.0e-06<br><br>5.0e-06	-	-	Q914G0; Y072_SIFVH Uncharacterized protein 72, *Sulfolobus islandicus* filamentous virus (isolate Iceland/Hveragerdi) (70.33/3.40e+00)<br><br>PF15533.9; Ntox33; Bacterial toxin 33 (90.44/2.1)	hp

				superfamily [*Myoviridae* sp.]					
33	27135	27890	+	putative antirepressor [uncultured *Caudovirales* phage]<br>regulatory protein [*Bacillus* phage phIS3501]<br>regulatory protein [*Bacillus* phage BtCS33]	7.0e-155<br>2.0e-95<br>9.0e-95	phage_pRha (13-115)<br>ORF6C (125-238)<br>Phage_pRha (22-109)	2.60e-34<br>3.37e-32<br>7.95e-32	P03037; RANT_BPP22 Antirepressor protein ant, Salmonella phage P22 (99.18/7.60e-09)<br>COG3646; pRha; Phage regulatory protein Rha [Mobilome: prophages, transposons] (98.72/1.20e-06)<br>COG3617; COG3617; Prophage antirepressor [Mobilome: prophages, transposons] (98.43/9.10e-05)	Rha-like protein (putative antirepressor)
34	27902	28090	+	hp	hp	-	-	COG4803; COG4803; Uncharacterized membrane protein [Function unknown] (76.59/7.20e+00)	hp

35	28117	28551	+	replication terminator protein [*AeriBacillus* phage AP45]<br><br>replication terminator protein [*Paenibacillus* phage Vegas]<br><br>replication terminator protein [*Paenibacillus* phage Dragolir]	2.0e-38<br><br>5.0e-28<br><br>8.0e-28	-	-	('-', '-')	putative replication terminator protein
36	28570	29283	+	hp	hp	-	-	COG5532; yfdQ; Uncharacterized conserved protein YfdQ, DUF2303 family [Function unknown] (95.14/3.20e+00)	hp
37	29283	29498	+	hp	hp	-	-	cd14652; Seven_helix_coil Seven_helix_coil. other structures are fusion proteins (84.2/9.80e-01)	hp
38	29864	30799	+	putative replication protein [uncultured *Caudovirales* phage]	0.0e+00	DnaD (144-287)	1.81e-13	COG3935; DnaD; DNA replication protein DnaD [Replication,	replication protein DnaD

				replication initiation protein [*Bacillus* phage BMBtp1]<br><br>DnaD domain protein [*Bacillus* phage phi4J1]	0.0e+00<br><br>1.0e-162	DnaD_dom<br>(184-245)	1.53e-11	recombination and repair] (99.16/5.10e-10)<br><br>P03688; VRPO_LAMBD Replication protein, *Escherichia* phage lambda (98.16/2.30e-06)<br><br>COG3611; DnaB; Replication initiation and membrane attachment protein DnaB [Replication, recombination and repair] (98.47/7.00e-06)	
39	30811	31290	+	hp	hp	-	-	P15854; GP166_BPPH5 Gene product 16.6, *Bacillus* phage phi15 (94.0/8.90e-03)<br><br>COG1675; TFA1; Transcription initiation factor IIE, alpha subunit [Transcription] (94.29/2.50e-02)	hp

								d1zina2; g.41.2.1 (A:126-160) Microbial and mitochondrial ADK, insert "zinc finger" domain {*Bacillus* stearothermophilus [TaxId: 1(91.48/3.90e-02)	
40	31283	31513	+	hp	hp	-	-	('-', '-')	hp
41	31537	32094	+	lysozyme-like protein [*Bacillus* phage vB_BtS_BMBtp14]<br><br>gp38 [*Bacillus* phage TP21-L]<br><br>TPA: MAG TPA: PVL ORF 50 like protein [*Myoviridae* sp.]	1.0e-83<br><br>2.0e-77<br><br>3.0e-23	-	-	Q6QGG9; D5_BPT5 Putative transcription factor D5, *Escherichia* phage T5 (93.82/4.90e-01)	hp
42	32133	32567	+	hp	hp	-	-	Q02406; Y12J_BPT4 Uncharacterized 7.3 kDa protein in Gp30-rIII intergenic region, *Enterobacteria* phage T4 (93.21/2.90e-01)	hp

43	32692	33228	+	putative dUTPase [uncultured *Caudovirales* phage]<br><br>dUTP diphosphatase [*Bacillus* phage vB_BtS_BMBtp3]<br><br>dUTPase [*Bacillus* phage vB_BthS-TP21T]	3.0e-115<br><br>4.0e-24<br><br>2.0e-22	dUTPase_2 (7-178)<br><br>NTP-PPase_dUTPase (11-93)<br><br>Dut2 (6-178)	2.99e-24<br><br>1.64e-18<br><br>2.78e-13	COG4508; Dut2; Dimeric dUTPase, all-alpha-NTP-PPase (MazG) superfamily [Nucleotide transport and metabolism] (100.0/1.70e-32)<br><br>d1w2ya_; a.204.1.1 (A:) Type II deoxyuridine triphosphatase {Campylobacter jejuni [TaxId: 197]} (99.96/1.00e-28)<br><br>d1ogla_; a.204.1.1 (A:) Type II deoxyuridine triphosphatase {Trypanosoma cruzi [TaxId: 5693]} (99.89/4.80e-22)	dUTPase diphosphatase
44	33271	33450	+	hp	hp	-	-	cd01716; Hfq; bacterial Hfq-like. Hfq, an abundant, ubiquitous RNA-binding protein, functions as a pleiotropic regulator of RNA m(93.28/3.90e-01)	hp
45	33491	34036	+	gp53 [*Listeria* phage A500]	5.0e-27	-	-	('-', '-')	hp

				gp40 [*Brochothrix* phage A9]	4.0e-25				
46	34058	34243	+	HNH endonuclease [*Bacillus* phage SWEP1]	1.0e-18	-	-	('-', '-')	hp
47	34259	34654	+	phage protein [*Bacillus* phage phIS3501]<br><br>TPA: MAG TPA: YopX protein [*Podoviridae* sp.]<br><br>TPA: MAG TPA: YopX protein [*Podoviridae* sp.]	7.0e-83<br><br>4.0e-26<br><br>2.0e-25	YopX<br>(5-130)<br><br>phage_TIGR01671<br>(5-59)	6.99e-12<br><br>4.14e-05	d2ox7a1; b.172.1.1 (A:4-145) Hypothetical protein EF1440 {*Enterococcus faecalis* [TaxId: 1351]} (99.94/8.30e-26)<br><br>d2p84a1; b.172.1.1 (A:4-135) Orf041 product {*Staphylococcus* phage 37 [TaxId: 320840]} (99.92/9.90e-25)<br><br>PF09643.13; YopX ; YopX protein (99.9.2.3e-23)	YopX protein domain-containing protein
48	34693	35091	+	putative structural protein [*Bacillus* phage P59]	1.0e-48	-	-	P13314; Y06G_BPT4 Uncharacterized 10.2 kDa protein in regB-denV intergenic region,	hp

				putative structural protein [*Bacillus* phage PK16]<br><br>putative structural protein [*Bacillus* phage BCP8-2]	4.0e-43<br><br>1.0e-42			*Enterobacteria* phage T4 (91.39/1.20e+00)	
49	35131	35439	+	hp	hp	-	-	cd00601; RNA_pol_P_RPB12; Eukaryotic RNA polymerases RPB12 subunit. RNA polymerases are multisubunit enzymes that synthesize RNA (70.63/4.30e+00)	hp
50	35474	35947	+	hp	hp	-	-	('-', '-')	hp
51	35982	36509	+	putative recombination protein U [uncultured *Caudovirales* phage]<br><br>Holliday junction resolvase RecU	4.0e-120<br><br>3.0e-89	RecU (8-168)	2.69e-61	COG3331; PrfA; Penicillin-binding protein-related factor A, putative recombinase [General function prediction only] (100.0/6.90e-36)<br><br>d1rzna_; c.52.1.28 (A:) Recombination protein U (RecU)/PBP related factor A (PrfA)	Holliday junction resolvase RecU

				[*Staphylococcus* phage SpaA1]<br><br>putative recombination protein U [*Bacillus* phage vB_BtS_BMBtp3]	2.0e-86			{*Bacillus subtilis* [TaxId: 1423]} (100.0/3.00e-34)<br><br>Q98VP9; HJC_SIRV1 Holliday junction resolvase, *Sulfolobus islandicus* rod-shaped virus 1 (99.16/2.80e-09)	
52	36506	36829	+	Zn-finger protein fused to HTH domain [*Staphylococcus* phage SpaA1]	1.0e-22	-	-	d1ijwc_; a.4.1.2 (C:) HIN recombinase (DNA-binding domain) {Synthetic}(97.62/4.40e-04)<br><br>d1gdta1; a.4.1.2 (A:141-183) gamma,delta resolvase (C-terminal domain) {*Escherichia coli* [TaxId: 562]}(97.52/7.20e-04)<br><br>d1rr7a_; a.4.1.14 (A:) Middle operon regulator, Mor {Bacteriophage Mu [TaxId: 10677]} (97.42/2.40e-03)	HTH domain containing protein

53	36965	37474	+	putative sigma-70 family RNA polymerase sigma factor [uncultured *Caudovirales* phage]<br>sigma-70 family RNA polymerase sigma factor [*Bacillus* phage Waukesha92]<br>positive control sigma-like factor [*Bacillus* phage phi4J1]	6.0e-98<br>4.0e-54<br>1.0e-53	PRK06930 (1-168)<br>sigma70-ECF (77-161)<br>Sigma70_r4 (104-157)	2.98e-60<br>2.92e-09<br>6.56e-09	COG4941; COG4941; Predicted RNA polymerase sigma factor, contains C-terminal TPR domain [Transcription] (99.65/4.00e-15)<br>P06227; RP34_BPSP1 RNA polymerase sigma GP34 factor, *Bacillus* phage SP01 (99.65/4.80e-15)<br>COG1595; RpoE; DNA-directed RNA polymerase specialized sigma subunit, sigma24 family [Transcription] (99.58/8.80e-14)	sigma-70 family RNA polymerase sigma factor
54	37892	38080	+	-	-	-	-	SCOP_d2heqa1 b.34.20.1 (A:7-71) Uncharacterized protein YorP {*Bacillus* subtilis [TaxId: 1423]} CLASS: All beta proteins, FOLD: SH3- (99.78/5.8e-18)	YorP-like protein

								2HEQ_A YorP protein; SH3-like, BSU2030, YorP, NESG, Structural Genomics, PSI-2, Protein Structure Initiative, Northeast Structu (99.59/3.3e-14)<br><br>Q02406 Y12J_BPT4 Uncharacterized 7.3 kDa protein in Gp30-rIII intergenic region OS=Enterobacteria phage T4 (99.11/3.5e-10)	
55	38147	39022	+	hp	hp	-	-	('-', '-')	hp
56	39513	39088	-	type II toxin-antitoxin system HicB family antitoxin [*AeriBacillus* phage AP45]<br><br>type II toxin-antitoxin system HicB family	5.0e-37	HicB_lk_antitox (8-127)<br><br>HicB (6-76)	2.88e-15<br><br>9.94e-10	P51716; YO14_BPHC1 Uncharacterized 14.9 kDa protein in rep-hol intergenic region, *Haemophilus* phage HP1 (strain HP1c1) (99.82/2.10e-18)<br><br>COG4226; HicB; Predicted nuclease of the RNAse H fold, HicB family	type II toxin-antitoxin system HicB family antitoxin

				antitoxin [*Clostridium* phage phiCDHM19]<br><br>type II toxin-antitoxin system HicB family antitoxin [*Bacillus* phage phi105]	1.0e-33<br><br>3.0e-33			[General function prediction only] (99.75/6.00e-17)<br><br>COG1598; HicB; Predicted nuclease of the RNAse H fold, HicB family [Defense mechanisms] (99.47/1.50e-12)	
57	39782	39591	-	TPA: MAG TPA: putative RNA binding protein [*Myoviridae* sp.]<br><br>TPA: MAG TPA: putative RNA-binding protein [*Myoviridae* sp.]<br><br>TPA: MAG TPA: putative RNA-binding protein [*Myoviridae* sp.]	3.0e-13<br><br>3.0e-13<br><br>2.0e-12	HicA_toxin (9-61)	1.95e-13	COG1724; YcfA; Predicted RNA binding protein YcfA, dsRBD-like fold, HicA-like mRNA interferase family [General function prediction (99.82/3.10e-19)<br><br>d1whza_; d.50.3.2 (A:) Hypothetical protein TTHA1913 {Thermus thermophilus [TaxId: 274]} (99.76/2.10e-17)	HicA-like family toxin

58	39953	40150	+	hp	hp	-	-	COG5443; FlbT; Flagellar biosynthesis regulator FlbT [Cell motility] (74.74/4.10e+00)	hp
59	40196	40489	+	hp	hp	-	-	d1xb2b3; d.43.1.1 (B:223-331) Elongation factor Ts (EF-Ts), dimerisation domain {Cow (*Bos taurus*), mitochondrial [TaxId: 9913]} (71.2/2.40e+00)	hp
60	40954	41154	+	-	-	-	-	('-', '-')	hp

Table S3. The large terminase subunit proteins of B450C and phages with well-studied DNA packaging mechanisms used for phylogenetic inference.

Terminus type		Phage	Number GenBank (terminase large subunit)	Source
**Exact direct terminal repeats**	Short direct terminal repeats (T7)	*Pseudomonas* phage Pf-10	YP_009145642.1	[3]
		Enterobacteria phage T7	QRE00040.1	[4]
		Enterobacteria phage T3	YP_009792972.1	[5]
		Yersinia phage phiYeO3-12	NP_052122.1	[6]
		Pseudomonas phage phi15	YP_004286227.1	[7]
		Pseudomonas phage PFP1	YP_009804025.1	[8]
	Short direct terminal repeats (N4)	*Escherichia phage* N4	YP_950546.1	[9]
		Achromobacter phage JWDelta	AHC56597.1	[10]
		Erwinia phage vB_EamP-S6	YP_007005834.1	[11]
	Short direct terminal repeats (c-st)	*Clostridium* phage c-st	YP_398598.1	[12]
		Bacillus phage Izhevsk	QIW89903.1	[13]
		Bacillus phage vB_BanS-Tsamsa	AGI11737.1	[14]
		Bacillus phage Basilisk	AGR46580.1	[15]
	Long DTRs (SPO1)	*Bacillus* virus SPO1	YP_002300330.1	[16]
		Listeria phage A511	YP_001468454.1	[17]
		Brochothrix phage A9	YP_004301396.1	[18]
	Long DTRs (T5)	*Enterobacteria* phage T5	YP_006983.1	[4]
		Salmonella virus SPC35	YP_004306624.1	[19]
		Providencia phage vB_PreS_PR1	YP_009599184.1	[20]
Cohesive ends	5′cos ends (lambda)	Bacteriophage N15	NP_046897.1	[21]
		Enterobacteria phage lambda	NP_040581.1	[4]
	5′cos ends (P2)	*Escherichia* virus P2	NP_046758.1	[4,22]
		Escherichia virus 186	NP_052251.1	[23]
		Pseudomonas phage phiCTX	NP_490600.1	[24]
	3′cos ends (HK97)	*Escherichia* virus HK97	NP_037698.1	[25]
		Escherichia virus HK022	NP_037663.1	[25]

		RHizobium phage 16-3	YP_002117560.1	[26]
		Bacillus phage B13	OP066531.2	[27]
**Host DNA at termini**	Host ends (Mu)	*Escherichia* virus Mu	AAF01106.1	[28]
		Pseudomonas phage B3	YP_164067.1	[29]
		Burkholderia virus BcepMu	YP_024701.1	[30]
	Host ends (D3112)	*Pseudomonas* virus MP22	YP_001469154.1	[31]
		Haemophilus phage SuMu	YP_007002934.1	[32]
		Pseudomonas virus D3112	NP_938233.1	[31]
**Circularly permuted direct terminal repeats**	Headful (P22)	*Salmonella* virus P22	YP_063734.1	[33]
		Salmonella phage ST64T	NP_720326.1	[34]
		Enterobacteria phage LP7	AAA88220.1	[35]
	Headful (Sf6)	*Shigella* phage Sf6	NP_958178.1	[36]
		Hamiltonella virus APSE-1	NP_050979.1	[37]
		Enterobacteria phage CUS-3	ABQ88401.1	[38]
	Headful (933W)	*Enterobacteria* phage 933W	NP_049511.1	[39]
		Burkholderia virus Bcep22	NP_944278.1	[40]
	Headful (phiKZ)	*Pseudomonas* phage phiKZ	NP_803591.1	[41]
		Pseudomonas phage 201phi2-1	YP_001956731.1	[42]
		Erwinia phage phiEaH2	YP_007237828.1	[43]
	Headful (T4)	*Enterobacteria* phage T4	NP_049776.1	[44]
		Vibrio phage KVP40	NP_899601.1	[44]
		Enterobacteria phage RB49	NP_891724.1	[45]
	Headful (phiPLPE)	*Yersinia* phage PY100	CAJ28416.1	[46]
		Klebsiella phage JD001	YP_007392855.1	[47]
		Iodobacterteriophage phiPLPE	YP_002128452.1	[48]
	Headful (SPP1)	*Bacillus* phage vB_BcM_Sam46	QIQ61203.1	[49]
		Bacillus phage SPP1	NP_690654.1	[50]
		Staphylococcus virus CNPH82	YP_950600.1	[51]
	Headful (B83)	*Bacillus* Phage vB_BtS_B83	QCQ57785.1	[52]
		Bacillus phage vB_BtS_BMBtp14	YP_009830709.1	[52]

	**Headful (B450)**	***Bacillus* phage B450T**	XYL31758.1	-
		Staphylococcus phage vB_SauS_Mh15	UKM36587.1	[53]
		Lactococcus phage proPhi6	QGJ84672.1	[54]
**Covalent terminal protein (phi29)**	Protein-primed mechanism [Longás,2008]	*Bacillus* phage phi29	YP_002004545.1	[55–57]
		Bacillus phage Nf	YP_009910733.1	[57]

Table S4. Phage genomes used for phylogenetic inference.

№	Name	Genome Accession number	Genome length	GC-content, %	SDCs	Number of tRNAs/tmRNAs	BLASTn nucleotide identity to B450T, %*	Proteins shared with B450T**	
								number	%
1	*Bacillus* phage B450T	PV695473.1	41,205	34.9	60	0/0	-	-	-
2	*Bacillus* phage B450C	PV695472.1	41,205	34.9	60	0/0	100	60	100
3	*Bacillus* phage B13	OP066531.2	36,864	34.8	53	0/0	37.80	24	42.5
4	*Bacillus* phage phi4J1	NC_029008.1	41,486	35.9	64	0/0	29.82	24	25.5
5	Uncultured *Caudovirales* phage clone 9AX_2, partial genome	MF417893.1	43,032	34.7	61	0/0	27.80	21	34.7
6	*Bacillus* phage BMBtp1	KT852578.1	35,838	34.9	58	0/0	21.74	19	32.2
7	*Bacillus* phage phiS58	KT970646.1	46,635	35.4	70	0/0	11.60	16	24.6
8	*Bacillus* phage vB_BthS-TP21T	MK843319.1	51,949	35.5	82	0/0	10.73	16	22.5
9	*Bacillus* phage BceA1	NC_048628.1	42,932	35.7	63	0/0	10.72	7	11.4
10	*Staphylococcus* phage SpaA1	NC_018277.1	42,784	35.6	65	0/0	10.72	7	11.2
11	*Bacillus* phage vB_BtS_BMBtp3	NC_028748.2	51,366	35.4	72	0/0	7.41	11	16.7
12	*Bacillus* phage phi4B1	NC_028886.1	38,663	35.9	64	0/0	6.60	7	12.96
13	*Bacillus anthracis* phage Cherry	DQ222851.1	36,615	35.3	53	0/0	7.84	7	12.4
14	*Bacillus* phage Gamma	NC_007458.1	37,253	35.2	55	0/0	7.90	7	12.2

15	*Bacillus anthracis* phage Gamma isolate d'Herelle	DQ289556.1	37,373	35.1	56	0/0	6.50	7	12.1
16	*Bacillus* phage AP631	MK085976.1	39,549	35.0	56	0/0	6.50	7	12.1
17	*Bacillus* phage WBeta	NC_007734.1	40,867	35.3	57	0/0	5.74	7	12.0
18	*Bacillus* phage Fah	NC_007814.1	37,974	34.9	54	0/0	7.14	6	10.5
19	*Bacillus* phage PfEFR-5	NC_031055.1	43,773	35.	68	0/0	7.40	6	9.4
20	*Bacillus* phage PfEFR-4	NC_048641.1	43,223	35.4	69	0/0	7.40	6	9.3
21	*Bacillus* phage phIS3501	NC_019502.1	44,401	34.9	69	1/0	11.48	5	7.8
22	*Bacillus* phage Waukesha92	NC_025424.1	45,648	35.5	71	0/0	12.40	4	6.1
23	*Bacillus* phage phiCM3	NC_023599.1	38,772	35.5	56	0/0	9.92	4	6.9
24	*Bacillus* phage BtCS33	NC_018085.1	41,992	35.2	59	0/0	8.26	4	6.7
25	*Bacillus* phage vB_BceS-MY192	NC_048633.1	44,696	35.0	66	0/0	6.65	4	6.4
26	*Bacillus* phage vB_BtS_B83	NC_048762.1	49,952	35.8	71	0/0	7.53	4	6.1
27	*Bacillus* phage TP21-L	NC_011645.1	37,456	37.	61	0/0	3.03	5	8.3
28	*Bacillus* phage vB_BtS_BMBtp2	NC_019912.1	36,932	37.8	55	0/0	2.79	4	7.0
29	*Bacillus* phage vB_BtS_BMBtp14	NC_048640.1	50,740	36.8	77	0/0	2.75	3	4.4
30	*Bacillus* phage vB_BanS_Athena	OK500002.1	37,369	35.3	62	0/0	0.80	3	5.0

31	*Bacillus* phage phBC6A51	NC_004820.1	61,395	37.7	94	0/0	4.10	1	1.3
32	*Bacillus* phage vB_BboS-125	NC_048735.1	58,528	48.6	87	0/0	0.00	1	1.4

*Determined using BLASTn compared to B450T phage (multiplying % coverage by % identity); **Determined using GET_HOMOLOGUES (COGtriangles algorithm, -G -t 0 -C 75). The additional six phage genomes found by the BLASTn search using the whole B450T genome sequence as the query are light gray.

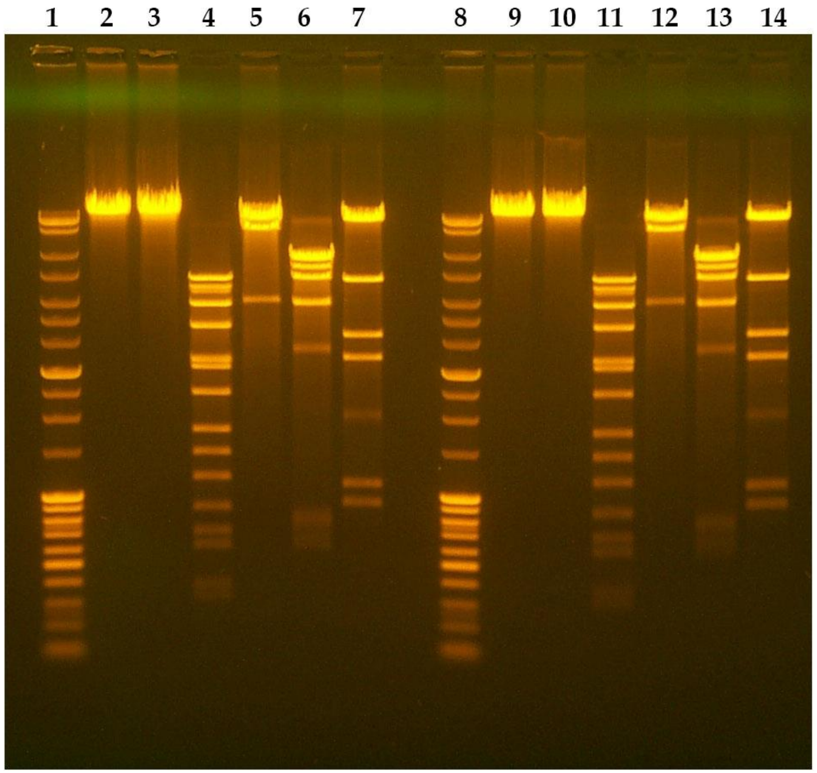

Figure S3. Restriction analysis of genomic DNA of B450C (1-7 lines) and B450T (8-14 lines) phages. This is the original gel image used to generate Figure 7, b in the main text. Kodak EDAS 290 Gel Documentation System ("Kodak") was used to capture the image. 1 and 8 – molecular weight markers; 2 and 9 – intact phage DNA; 3 and 10 – NotI; 4 and 11 – HindIII; 5 and 12 – BamHI; 6 and 13 – BglII; 7 and 14.

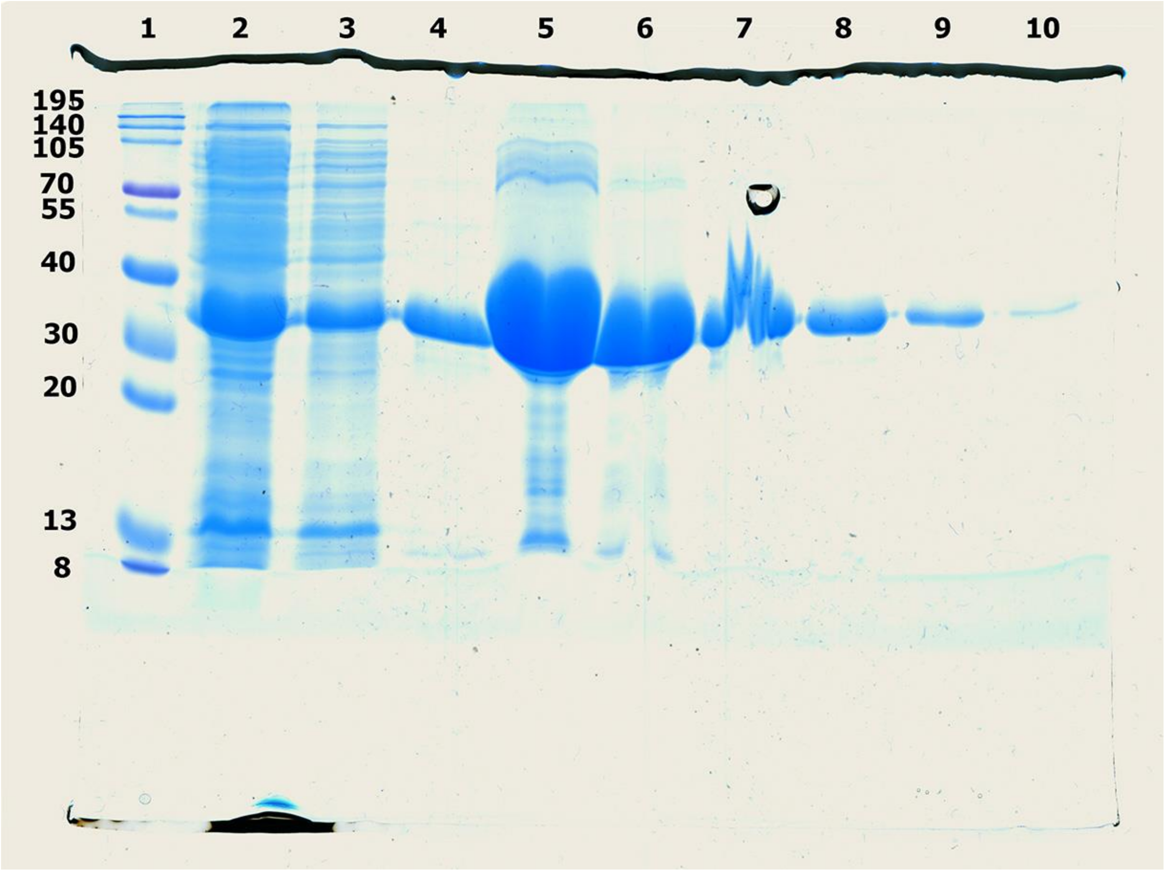

Figure S4. Coomassie brilliant blue R-250-stained SDS–PAGE gel illustrating the purification of recombinant PlyC19-His_6 endolysin by Ni^{2+}-nitrilotriacetic acid immobilized metal affinity chromatography (Ni-NTA IMAC). **1** – Prestained Protein Marker VII (Servicebio; 8–195 kDa); **2** – flow-through; **3** – wash with buffer A; **4** – wash with buffer B + 20 mM imidazole; **5-9** – elution fractions with buffer B + 250 mM imidazole (the arrowhead indicates the position of the target protein at ≈35.8 kDa); **10** – stripping with buffer B + 1 M imidazole.

Table S5. Endolysins of bacteriophages infecting the genus *Bacillus*, used to construct a phylogenetic tree. Abbreviations: EAD – enzymatically active domain, CBD – cell-wall binding domain.

№	Genome Accession number	Genome name	EAD	CBD	Protein Accession number
1	MF418016.1	*Bacillus* phage vB_BceM-HSE3	Amidase_2	2xSH3	AWD93052.1
2	KX227759.1	*Bacillus* phage PfIS075	Amidase_2	-	ANT40327.1
3	MT745955.1	*Bacillus* phage J5a	Amidase_2	-	YP_010739914.1
4	MT745954.2	*Bacillus* phage F16Ba	Amidase_2	-	YP_010739622.1
5	MG727702.1	*Paenibacillus* phage Likha	Amidase_2	-	YP_009836640.1
6	MG727695.1	*Paenibacillus* phage BN12	Amidase_2	-	YP_009836278.1
7	MG727696.1	*Paenibacillus* phage Kiel007	Amidase_2	-	AUS03644.1
8	MG727698.1	*Paenibacillus* phage PBL1c	Amidase_2	-	YP_009836351.1
9	MG727700.1	*Paenibacillus* phage Tadhana	Amidase_2	-	YP_009836500.1
10	MG727699.1	*Paenibacillus* phage Pagassa	Amidase_2	-	YP_009836430.1
11	MG727701.1	*Paenibacillus* phage Leyra	Amidase_2	-	AUS03845.1
12	MG727697.1	*Paenibacillus* phage Dragolir	Amidase_2	-	YP_010080174.1
13	OP066531.2	*Bacillus* phage B13	Amidase_2	2xSH3	UUW40205.1
14	PV695473.1	*Bacillus* phage B450T	Amidase_2	2xSH3	XYL31778.1
15	KJ920400.1	*Bacillus* phage Waukesha92	Amidase_2	2xSH3	YP_009099314.1
16	MK843319.1	*Bacillus* phage vB_BthS-TP21T	Amidase_2	2xSH3	QCW20859.1
17	KT970646.1	*Bacillus* phage phiS58	Amidase_2	2xSH3	ALO79948.1
18	KF296718.1	*Bacillus* phage phiCM3	Amidase_2	SH3	YP_009009166.1
19	MN065183.1	*Bacillus* phage vB_BthS-HD29phi	Amidase_2	SH3	QDP43493.1
20	NC_019502.1	*Bacillus* phage phIS3501	Amidase_2	SH3	37632-38273 bp*
21	OQ317942.1	*Bacillus* phage 0105phi7-2	Amidase_2	CBD_PlyG	YP_010742694.1

22	MG584725.1	*Bacillus* phage BVE2	Amidase_2	CBD_PlyG	AUG88606.1
23	DQ150593.1	*Bacillus* phage Fah	Amidase_2	CBD_PlyG	ABA42708.1
24	DQ222853.1	*Bacillus* anthracis phage (Gamma isolate 51)	Amidase_2	CBD_PlyG	ABA46449.1
25	DQ222851.1	*Bacillus* anthracis phage Cherry	Amidase_2	CBD_PlyG	ABA46392.1
26	DQ289555.1	*Bacillus* phage Wbeta	Amidase_2	CBD_PlyG	ABC40416.1
27	MK085976.1	*Bacillus* phage AP631	Amidase_2	CBD_PlyG	YP_010739459.1
28	DQ289556.1	*Bacillus* anthracis phage (Gamma isolate d'Herelle)	Amidase_2	CBD_PlyG	ABC40469.1
29	KX227757.1	*Bacillus* phage PfEFR-4	Amidase_2	CBD_PlyG	ANT40164.1
30	GU229986.1	*Bacillus* phage 250	Amidase_2	CBD_PlyG	ADB28383.1
31	MH458951.1	*Bacillus* phage vB_BthS_BMBphi	Amidase_2	-	AXF39889.1
32	JN654439.1	*Bacillus* phage BPS13	Amidase_2	SH3	YP_006907567.1
33	KU737344.1	*Bacillus* phage Nigalana	Amidase_2	SH3	YP_009282466.1
34	KT070867.1	*Bacillus* phage PBC2	Amidase_2	2xSH3	YP_010679375.1
35	MT254578.1	*Bacillus* phage Izhevsk	Amidase_2	2xSH3	YP_010680601.1
36	MK288021.1	*Bacillus* phage pW2	Amidase_2	2xSH3	YP_010680031.1

* Location of the gene encoding the endolysin in the phage genome (if no protein accession number is available).

Table S6. Reagent list.

No.	Reagent	Company name, address (city, country)/ other information
1	Acetic Acid	Kupavnareaktiv, Staraya Kupavna, Russia / chemically pure
2	Agar	Dia-M, Moscow, Russia / bacteriology grade, Cat.No. 3346.0500
3	Agarose	Bio-Rad, Richmond, CA, USA / standard electroendosmosis
4	Gelatin	Difco laboratories, Detroit, MI, USA
5	Glycine	SERVA, Heidelberg, Germany / analytical grade
6	Calcium Chloride	Dalchempharm, Khabarovsk, Russia / pharm. grade
7	Cesium Chloride	Reachim, Staraya Kupavna, Russia / high purity grade
8	Magnesium Chloride	Aldosa, Moscow, Russia / chemically pure
9	PEG 8000	PanReac AppliChem, Darmstadt, Germany / pharm. grade
10	Potassium chloride	Dia-M, Moscow, Russia / pharm. grade
11	Hydrochloric Acid	Kupavnareaktiv, Staraya Kupavna, Russia / chemically pure
12	Magnesium Sulphate	Labochem international, Einhausen, Germany / pharm. grade
13	Sodium Acetate	PanReac, Darmstadt Germany / pharm. grade
14	Sodium Chloride	Kupavnareaktiv, Staraya Kupavna, Russia / chemically pure
15	Sodium Hydroxide	PanReac AppliChem, Darmstadt, Germany / pharm. grade
16	Sodium Phosphate, dibasic	PanReac, Darmstadt, Germany / pharm. grade
17	Sodium Phosphate, monobasic	PanReac, Darmstadt, Germany / pharm. grade
18	Tris(hydroxymethyl)aminomethane	Sigma, Burlington, Massachusetts, United States / BioXtra
19	Trypton	Dia-M, Moscow, Russia / Cat.No. 3313.0500
20	Yeast Extract	Dia-M, Moscow, Russia / Cat.No. 3017.0500

References

1. Kazantseva, O.A.; Kulyabin, V.A.; Koposova, O.N.; Shadrin, A.M. The Genome Sequence of *Bacillus Toyonensis* Strain IP 5832 Isolated from the Probiotic "Bactisubtil" Contains Genes of *Bacillus Cereus* Pathogenicity Factors and Antibiotic Resistance. *Microbiology (Russian Federation)* **2024**, *93*, 973–981, doi:10.1134/S0026261724606870.

2. Sorokin, A.; Candelon, B.; Guilloux, K.; Galleron, N.; Wackerow-Kouzova, N.; Ehrlich, S.D.; Bourguet, D.; Sanchis, V. Multiple-locus sequence typing analysis of *Bacillus cereus* and *Bacillus thuringiensis* reveals separate clustering and a distinct population structure of psychrotrophic strains. *Appl Environ Microbiol* **2006**, *72*, 1569–1578, doi:10.1128/AEM.72.2.1569-1578.2006.

3. Kazantseva, O.A.; Buzikov, R.M.; Pilipchuk, T.A.; Valentovich, L.N.; Kazantsev, A.N.; Kalamiyets, E.I.; Shadrin, A.M. The Bacteriophage Pf-10 – a component of the biopesticide "Multiphage" used to control agricultural crop diseases caused by *Pseudomonas syringae*. *Viruses* **2022**, *14*, 42, doi:10.3390/v14010042.

4. Casjens, S.R.; Gilcrease, E.B. Determining DNA packaging strategy by analysis of the termini of the chromosomes in tailed-bacteriophage virions. *Methods Mol Biol* **2009**, *502*, 91–111, doi:10.1007/978-1-60327-565-1_7.

5. Pajunen, M.I.; Elizondo, M.R.; Skurnik, M.; Kieleczawa, J.; Molineux, I.J. Complete Nucleotide Sequence and Likely Recombinatorial Origin of Bacteriophage T3. *J Mol Biol* **2002**, *319*, 1115–1132, doi:10.1016/S0022-2836(02)00384-4.

6. Pajunen, M.; Kiljunen, S.; Skurnik, M. Bacteriophage ΦYeO3-12, Specific for *Yersinia Enterocolitica* Serotype O:3, Is Related to Coliphages T3 and T7. *J Bacteriol* **2000**, *182*, doi:10.1128/JB.182.18.5114-5120.2000.

7. Cornelissen, A.; Ceyssens, P.J.; T'Syen, J.; van Praet, H.; Noben, J.P.; Shaburova, O. V.; Krylov, V.N.; Volckaert, G.; Lavigne, R. The T7-Related *Pseudomonas Putida* Phage Φ15 Displays Virion-Associated Biofilm Degradation Properties. *PLoS One* **2011**, *6*, doi:10.1371/journal.pone.0018597.

8. Li, M.; Chen, X.; Ma, Y.; Li, Z.; Zhao, Q. Complete Genome Sequence of PFP1, a Novel T7-like *Pseudomonas Fluorescens* Bacteriophage. *Arch Virol* **2018**, *163*, doi:10.1007/s00705-018-3979-3.

9. Wittmann, J.; Turner, D.; Millard, A.D.; Mahadevan, P.; Kropinski, A.M.; Adriaenssens, E.M. From Orphan Phage to a Proposed New Family–the Diversity of N4-like Viruses. *Antibiotics* **2020**, *9*, doi:10.3390/antibiotics9100663.

10. Ma, Y.; Li, E.; Qi, Z.; Li, H.; Wei, X.; Lin, W.; Zhao, R.; Jiang, A.; Yang, H.; Yin, Z.; et al. Isolation and Molecular Characterisation of *Achromobacter* Phage PhiAxp-3, an N4-like Bacteriophage. *Sci Rep* **2016**, *6*, doi:10.1038/srep24776.

11. Born, Y.; Fieseler, L.; Marazzi, J.; Lurz, R.; Duffy, B.; Loessner, M.J. Novel Virulent and Broad-Host-Range *Erwinia Amylovora* Bacteriophages Reveal a High Degree of Mosaicism and a Relationship to *Enterobacteriaceae* Phages. *Appl Environ Microbiol* **2011**, *77*, doi:10.1128/AEM.03022-10.

12. Sakaguchi, Y.; Hayashi, T.; Kurokawa, K.; Nakayama, K.; Oshima, K.; Fujinaga, Y.; Ohnishi, M.; Ohtsubo, E.; Hattori, M.; Oguma, K. The genome sequence of *Clostridium botulinum* type C neurotoxin-converting phage and the molecular mechanisms of unstable lysogeny. *Proc Natl Acad Sci U S A* **2005**, *102*, 17472–17477, doi:10.1073/pnas.0505503102.

13. Skorynina, A. V.; Piligrimova, E.G.; Kazantseva, O.A.; Kulyabin, V.A.; Baicher, S.D.; Ryabova, N.A.; Shadrin, A.M. *Bacillus*-infecting bacteriophage Izhevsk harbors thermostable endolysin with broad range specificity. *PLoS One* **2020**, *15*, e0242657, doi:10.1371/journal.pone.0242657.

14. Ganz, H.H.; Law, C.; Schmuki, M.; Eichenseher, F.; Calendar, R.; Loessner, M.J.; Getz, W.M.; Korlach, J.; Beyer, W.; Klumpp, J. Novel giant siphovirus from *Bacillus anthracis* features unusual genome characteristics. *PLoS One* **2014**, *9*, e85972, doi:10.1371/journal.pone.0085972.

15. Grose, J.H.; Jensen, J.D.; Merrill, B.D.; Fisher, J.N.B.; Burnett, S.H.; Breakwell, D.P. Genome Sequences of Three Novel *Bacillus Cereus* Bacteriophages. *Genome Announc* **2014**, *2*, doi:10.1128/genomea.01118-13.

16. Stewart, C.R.; Casjens, S.R.; Cresawn, S.G.; Houtz, J.M.; Smith, A.L.; Ford, M.E.; Peebles, C.L.; Hatfull, G.F.; Hendrix, R.W.; Huang, W.M.; et al. The genome of *Bacillus subtilis* Bacteriophage SPO1. *J Mol Biol* **2009**, *388*, 48–70, doi:10.1016/j.jmb.2009.03.009.

17. Klumpp, J.; Dorscht, J.; Lurz, R.; Bielmann, R.; Wieland, M.; Zimmer, M.; Calendar, R.; Loessner, M.J. The Terminally Redundant, Nonpermuted Genome of *Listeria* Bacteriophage A511: A Model for the SPO1-like Myoviruses of Gram-Positive Bacteria. *J Bacteriol* **2008**, *190*, doi:10.1128/JB.00461-08.

18. Kilcher, S.; Loessner, M.J.; Klumpp, J. *Brochothrix Thermosphacta* Bacteriophages Feature Heterogeneous and Highly Mosaic Genomes and Utilize Unique Prophage Insertion Sites. *J Bacteriol* **2010**, *192*, doi:10.1128/JB.00709-10.

19. Kim, M.; Ryu, S. Characterization of a T5-like Coliphage, SPC35, and Differential Development of Resistance to SPC35 in *Salmonella Enterica* Serovar *Typhimurium* and *Escherichia Coli*. *Appl Environ Microbiol* **2011**, *77*, doi:10.1128/AEM.02504-10.

20. Oliveira, H.; Pinto, G.; Hendrix, H.; Noben, J.-P.; Gawor, J.; Kropinski, A.M.; Łobocka, M.; Lavigne, R.; Azeredo, J. A Lytic Providencia Rettgeri Virus of Potential Therapeutic Value Is a Deep-Branching Member of the T5virus Genus. *Appl Environ Microbiol* **2017**, *83*, doi:10.1128/AEM.01567-17.

21. Ravin, N. V. N15: The Linear Phage-Plasmid. *Plasmid* 2011, *65*.

22. Christie, G.E.; Calendar, R. P2 Bacteriophage. *Bacteriophage* **2016**, *6*, doi:10.1080/21597081.2016.1145782.

23. Bullas, L.R.; Mostaghimi, A.R.; Arensdorf, J.J.; Rajadas, P.T.; Zuccarelli, A.J. *Salmonella* Phage PSP3, Another Member of the P2-like Phage Group. *Virology* **1991**, *185*, doi:10.1016/0042-6822(91)90573-T.

24. Nakayama, K.; Kanaya, S.; Ohnishi, M.; Terawaki, Y.; Hayashi, T. The Complete Nucleotide Sequence of PhiCTX, a Cytotoxin-Converting Phage of *Pseudomonas Aeruginosa*: Implications for Phage Evolution and Horizontal Gene Transfer via Bacteriophages. *Mol Microbiol* **1999**, *31*, 399–419, doi:10.1046/j.1365-2958.1999.01158.x.

25. Juhala, R.J.; Ford, M.E.; Duda, R.L.; Youlton, A.; Hatfull, G.F.; Hendrix, R.W. Genomic sequences of bacteriophages HK97 and HK022: pervasive genetic mosaicism in the lambdoid bacteriophages. *J Mol Biol* **2000**, *299*, 27–51, doi:10.1006/jmbi.2000.3729.

26. Ganyu, A.; Csiszovszki, Z.; Ponyi, T.; Kern, A.; Buzás, Z.; Orosz, L.; Papp, P.P. Identification of Cohesive Ends and Genes Encoding the Terminase of Phage 16-3. *J Bacteriol* **2005**, *187*, doi:10.1128/JB.187.7.2526-2531.2005.

27. Kazantseva, O.A.; Piligrimova, E.G.; Shadrin, A.M. Novel *Bacillus*-infecting bacteriophage B13 – the founding member of the proposed new genus *Bunatrivirus*. *Viruses* **2022**, *14*, 2300, doi:10.3390/v14102300.

28. Morgan, G.J.; Hatfull, G.F.; Casjens, S.; Hendrix, R.W. Bacteriophage Mu genome sequence: analysis and comparison with Mu-like prophages in *Haemophilus*, *Neisseria* and *Deinococcus*. *J Mol Biol* **2002**, *317*, 337–359, doi:10.1006/jmbi.2002.5437.

29. Braid, M.D.; Silhavy, J.L.; Kitts, C.L.; Cano, R.J.; Howe, M.M. Complete Genomic Sequence of Bacteriophage B3, a Mu-like Phage of *Pseudomonas Aeruginosa*. *J Bacteriol* **2004**, *186*, doi:10.1128/JB.186.19.6560-6574.2004.

30. Summer, E.J.; Gonzalez, C.F.; Carlisle, T.; Mebane, L.M.; Cass, A.M.; Savva, C.G.; LiPuma, J.J.; Young, R. *Burkholderia Cenocepacia* Phage BcepMu and a Family of Mu-like Phages Encoding Potential Pathogenesis Factors. *J Mol Biol* **2004**, *340*, doi:10.1016/j.jmb.2004.04.053.

31. Heo, Y.J.; Chung, I.Y.; Choi, K.B.; Lau, G.W.; Cho, Y.H. Genome Sequence Comparison and Superinfection between Two Related *Pseudomonas Aeruginosa* Phages, D3112 and MP22. *Microbiology (N Y)* **2007**, *153*, doi:10.1099/mic.0.2007/007260-0.

32. Zehr, E.S.; Tabatabai, L.B.; Bayles, D.O. Genomic and Proteomic Characterization of SuMu, a Mu-like Bacteriophage Infecting *Haemophilus Parasuis*. *BMC Genomics* **2012**, *13*, doi:10.1186/1471-2164-13-331.

33. Byl, C. V.; Kropinski, A.M. Sequence of the Genome of *Salmonella* Bacteriophage P22. *J Bacteriol* **2000**, *182*, doi:10.1128/JB.182.22.6472-6481.2000.

34. Mmolawa, P.T.; Schmieger, H.; Tucker, C.P.; Heuzenroeder, M.W. Genomic Structure of the *Salmonella Enterica* Serovar *Typhimurium* DT 64 Bacteriophage ST64T: Evidence for Modular Genetic Architecture. *J Bacteriol* **2003**, *185*, doi:10.1128/JB.185.11.3473-3475.2003.

35. Bernhard Petri, J.; Schmieger, H. Isolation of Fragments with Pac Function for Phage P22 from Phage LP7 DNA and Comparison of Packaging Gene 3 Sequences. *Gene* **1990**, *88*, doi:10.1016/0378-1119(90)90058-Y.

36. Zhao, H.; Christensen, T.E.; Kamau, Y.N.; Tang, L. Structures of the Phage Sf6 Large Terminase Provide New Insights into DNA Translocation and Cleavage. *Proc Natl Acad Sci U S A* **2013**, *110*, doi:10.1073/pnas.1301133110.

37. Van Der Wilk, F.; Dullemans, A.M.; Verbeek, M.; Van Den Heuvel, J.F.J.M. Isolation and Characterization of APSE-1, a Bacteriophage Infecting the Secondary Endosymbiont of *Acyrthosiphon Pisum*. *Virology* **1999**, *262*, doi:10.1006/viro.1999.9902.

38. King, M.R.; Vimr, R.P.; Steenbergen, S.M.; Spanjaard, L.; Plunkett, G.; Blattner, F.R.; Vimr, E.R. *Escherichia Coli* K1-Specific Bacteriophage CUS-3 Distribution and Function in Phase-Variable Capsular Polysialic Acid O Acetylation. *J Bacteriol* **2007**, *189*, doi:10.1128/JB.00657-07.

39. Plunkett, G.; Rose, D.J.; Durfee, T.J.; Blattner, F.R. Sequence of Shiga toxin 2 phage 933W from *Escherichia coli* O157:H7: Shiga toxin as a phage late-gene product? *J Bacteriol* **1999**, *181*, 1767–1778, doi:10.1128/jb.181.6.1767-1778.1999.

40. Gill, J.J.; Summer, E.J.; Russell, W.K.; Cologna, S.M.; Carlile, T.M.; Fuller, A.C.; Kitsopoulos, K.; Mebane, L.M.; Parkinson, B.N.; Sullivan, D.; et al. Genomes and Characterization of Phages Bcep22 and BcepIL02, Founders of a Novel Phage Type in *Burkholderia Cenocepacia*. *J Bacteriol* **2011**, *193*, doi:10.1128/JB.05287-11.

41. Lecoutere, E.; Ceyssens, P.J.; Miroshnikov, K.A.; Mesyanzhinov, V. V.; Krylov, V.N.; Noben, J.P.; Robben, J.; Hertveldt, K.; Volckaert, G.; Lavigne, R. Identification and Comparative Analysis of the Structural Proteomes of ΦKZ and EL, Two Giant *Pseudomonas Aeruginosa* Bacteriophages. *Proteomics* **2009**, *9*, doi:10.1002/pmic.200800727.

42. Thomas, J.A.; Rolando, M.R.; Carroll, C.A.; Shen, P.S.; Belnap, D.M.; Weintraub, S.T.; Serwer, P.; Hardies, S.C. Characterization of *Pseudomonas Chlororaphis* Myovirus 201ϕ{symbol}2-1 via Genomic Sequencing, Mass Spectrometry, and Electron Microscopy. *Virology* **2008**, *376*, doi:10.1016/j.virol.2008.04.004.

43. Dömötör, D.; Becságh, P.; Rákhely, G.; Schneider, G.; Kovács, T. Complete Genomic Sequence of *Erwinia Amylovora* Phage PhiEaH2. *J Virol* **2012**, *86*, doi:10.1128/jvi.01870-12.

44. Miller, E.S.; Heidelberg, J.F.; Eisen, J.A.; Nelson, W.C.; Durkin, A.S.; Ciecko, A.; Feldblyum, T. V.; White, O.; Paulsen, I.T.; Nierman, W.C.; et al. Complete Genome Sequence of the Broad-Host-Range Vibriophage KVP40: Comparative Genomics of a T4-Related Bacteriophage. *J Bacteriol* **2003**, *185*, doi:10.1128/JB.185.17.5220-5233.2003.

45. Desplats, C.; Dez, C.; Tétart, F.; Eleaume, H.; Krisch, H.M. Snapshot of the Genome of the Pseudo-T-Even Bacteriophage RB49. *J Bacteriol* **2002**, *184*, doi:10.1128/JB.184.10.2789-2804.2002.

46. Schwudke, D.; Ergin, A.; Michael, K.; Volkmar, S.; Appel, B.; Knabner, D.; Konietzny, A.; Strauch, E. Broad-Host-Range *Yersinia* Phage PY100: Genome Sequence, Proteome Analysis of Virions, and DNA Packaging Strategy. *J Bacteriol* **2008**, *190*, doi:10.1128/JB.01402-07.

47. Cui, Z.; Shen, W.; Wang, Z.; Zhang, H.; Me, R.; Wang, Y.; Zeng, L.; Zhu, Y.; Qin, J.; He, P.; et al. Complete Genome Sequence of *Klebsiella Pneumoniae* Phage JD001. *J Virol* **2012**, *86*, doi:10.1128/jvi.02435-12.

48. Leblanc, C.; Caumont-Sarcos, A.; Comeau, A.M.; Krisch, H.M. Isolation and Genomic Characterization of the First Phage Infecting Iodobacteria: ΦPLPE, a Myovirus Having a Novel Set of Features. *Environ Microbiol Rep* **2009**, *1*, doi:10.1111/j.1758-2229.2009.00055.x.

49. Kazantseva, O.A.; Piligrimova, E.G.; Shadrin, A.M. vB_BcM_Sam46 and vB_BcM_Sam112, members of a new bacteriophage genus with unusual small terminase structure. *Sci Rep* **2021**, *11*, 12173, doi:10.1038/s41598-021-91289-x.

50. Martínez-Jiménez, M.I.; Alonso, J.C.; Ayora, S. *Bacillus subtilis* bacteriophage SPP1-encoded gene 34.1 product is a recombination-dependent DNA replication protein. *J Mol Biol* **2005**, *351*, 1007–1019, doi:10.1016/j.jmb.2005.06.064.

51. Daniel, A.; Bonnen, P.E.; Fischetti, V.A. First Complete Genome Sequence of Two *Staphylococcus Epidermidis* Bacteriophages. *J Bacteriol* **2007**, *189*, doi:10.1128/JB.01637-06.

52. Piligrimova, E.G.; Kazantseva, O.A.; Nikulin, N.A.; Shadrin, A.M. *Bacillus* phage vB_BtS_b83 previously designated as a plasmid may represent a new *Siphoviridae* genus. *Viruses* **2019**, *11*, 624, doi:10.3390/v11070624.

53. Suárez, C.A.; Carrasco, S.T.; Brandolisio, F.N.A.; Abatangelo, V.; Boncompain, C.A.; Peresutti-Bacci, N.; Morbidoni, H.R. Bioinformatic Analysis of a Set of 14 Temperate Bacteriophages Isolated from *Staphylococcus Aureus* Strains Highlights Their Massive Genetic Diversity. *Microbiol Spectr* **2022**, *10*, doi:10.1128/spectrum.00334-22.

54. Alexeeva, S.; Liu, Y.; Zhu, J.; Kaczorowska, J.; Kouwen, T.R.H.M.; Abee, T.; Smid, E.J. Genomics of Tailless Bacteriophages in a Complex Lactic Acid Bacteria Starter Culture. *Int Dairy J* **2021**, *114*, doi:10.1016/j.idairyj.2020.104900.

55. Simpson, A.A.; Tao, Y.; Leiman, P.G.; Badasso, M.O.; He, Y.; Jardine, P.J.; Olson, N.H.; Morais, M.C.; Grimes, S.; Anderson, D.L.; et al. Structure of the Bacteriophage Φ29 DNA Packaging Motor. *Nature* **2000**, *408*, doi:10.1038/35047129.

56. Morais, M.C.; Koti, J.S.; Bowman, V.D.; Reyes-Aldrete, E.; Anderson, D.L.; Rossmann, M.G. Defining Molecular and Domain Boundaries in the Bacteriophage Φ29 DNA Packaging Motor. *Structure* **2008**, *16*, doi:10.1016/j.str.2008.05.010.

57. Longás, E.; Villar, L.; Lázaro, J.M.; De Vega, M.; Salas, M. Phage Φ29 and Nf Terminal Protein-Priming Domain Specifies the Internal Template Nucleotide to Initiate DNA Replication. *Proc Natl Acad Sci U S A* **2008**, *105*, doi:10.1073/pnas.0809882105.
